# Comparison of DNA extraction methods on CITES-listed timber species and application in species authentication of commercial products using DNA barcoding

**DOI:** 10.1038/s41598-022-27195-7

**Published:** 2023-01-04

**Authors:** Grace Wing-Chiu But, Hoi-Yan Wu, Tin-Yan Siu, Kwun-Tin Chan, Kwan-Ho Wong, David Tai-Wai Lau, Pang-Chui Shaw

**Affiliations:** 1 School of Life Sciences, The Chinese University of Hong Kong, Hong Kong, China; 2 Li Dak Sum Yip Yio Chin R & D Centre for Chinese Medicine, The Chinese University of Hong Kong, Hong Kong, China; 3 Shiu-Ying Hu Herbarium, School of Life Sciences, The Chinese University of Hong Kong, Hong Kong, China; 4 Research Area of Ecology and Biodiversity, School of Biological Sciences, The University of Hong Kong, Hong Kong, China; 5 State Key Laboratory of Research on Bioactivities and Clinical Applications of Medicinal Plants (The Chinese University of Hong Kong) and Institute of Chinese Medicine, The Chinese University of Hong Kong, Hong Kong, China

**Keywords:** DNA sequencing, DNA sequencing, DNA

## Abstract

Quality and quantity of DNA extracted from wood is important for molecular identification of wood species, which can serve for conservation of wood species and law enforcement to combat illegal wood trading. Rosewood (*Dalbergia* and *Pterocarpus*) and agarwood (*Aquilaria*) are the most commonly found hardwood in timber seizure incidents. To monitor international trade of timber and commercial wood products and to protect these endangered wood species from further population decline, in this study, we have compared three extraction protocols for DNA extraction from 12 samples of rosewood and agarwood timber logs, and later applied the best DNA extraction protocol on 10 commercial wood products claimed to be rosewood and agarwood. We also demonstrated the applicability of DNA mini-barcoding with multi-loci combination with reference library for identifying the species of timber and commercial wood products. We found that a silica column-based method with guanidine thiocyanate-containing binding buffer served the best in DNA extraction from different parts of wood in all three genera with good quality and quantity. Single barcode region ITS2 or multi-loci combinations including ITS2 barcode region generally provide better discriminatory power for species identification for both rosewood and agarwood. All 10 products were identified to species-level using multi-loci combination. In terms of accuracy in labelling, 80% of them were labelled correctly. Our work has shown the feasibility of extracting good quality of DNA from authentic wood samples and processed wood products and identifying them to species level based on DNA barcoding technology.

## Introduction

Increasing demand of high-value hardwood timber in the last 20 years has driven illegal logging and illegal timber trade. “Rosewood” and “agarwood” are the most commonly found hardwood in seizure incidents. The United Nations estimated that seizures of “rosewood” and “agarwood” account for the largest share, 35% (around 8 million kg) and 6% of total wildlife seizures, respectively, in 2005–2014^1^. “Rosewood” is a commercial name representing a range of tropical hardwood species which are mostly traded for high-end furniture, carving, and accessory manufacture. Species of the genus *Dalbergia* and *Pterocarpus* are not only the source plants of rosewoods listed in the appendices of *Convention on International Trade in Endangered Species of Wild Fauna and Flora* (CITES)^2^, but also the source plants of “*Hongmu*” listed under the *National Standards of the People’s Republic of China for Hongmu* (GB/T 18107-2017)^3^ which are used for making rosewood products^4^. On the other hand, agarwood is a fragrant resinous wood formed in the heartwood of *Aquilaria* species due to fungal infection^5^. Agarwood is mostly traded as incense or wood piece for its fragrance with a high commercial value.

As a free port next to mainland China, Hong Kong has become a convenient gateway for illegal import and smuggling of timber logs and products of rosewood and agarwood^6^. It was estimated that around 30% of timber imported into Hong Kong in 2007 were from illegal sources^7^. In 2017–2021, Hong Kong Customs have seized more than 365,000 kg of rosewood timber, with a total estimated market value of over HKD 100 million^8^. In addition to rosewood trafficking, illegal felling of agar trees is another common timber crime in Hong Kong. Increasing number of transborder itinerant theft from mainland China have been found from 2005–2013^9^. Illegal logging is a highly destructive wildlife crime, as it threatens both the targeted tree species and the entire habitat along with decline in biological diversity.

In order to protect these tree species from overexploitation, CITES has listed agarwood species (*Aquilaria* spp.) and some of the rosewood species including *Dalbergia* and *Pterocarpus* spp*.* in Appendix I and II to ensure legal and sustainable trade of timber and wood products. To enforce the CITES obligations and identify illegal trade, accurate wood identification is necessary. Traditional wood anatomy based on the macroscopic and microscopic anatomical features of timber has been widely established for wood identification^10,11^. Molecular methods such as DNA barcoding and real-time PCR, have also been developed for identification of rosewood and agarwood, mostly to genus-level^12–17^. Recent studies have shown the use of DNA barcoding with multi-loci combinations with reference database could provide a better discriminatory power and allow species-level identification for *Dalbergia* (ITS2 + *trnH-psbA*)^18^, *Pterocarpus* (*matK* + *ndhF*-*rpl32* + ITS2)^19^, and *Aquilaria* (*trnL*-*trnF* + ITS2)^20^.

To achieve successful wood identification using molecular techniques, it is important to obtain sufficient good-quality, amplifiable DNA from wood samples. However, it is difficult to extract DNA from dried, aged timber heartwood, let alone highly processed commercial wood products. DNA isolated from wood usually contains high levels of tannins, phenolic and lignin compounds, which inhibit subsequent molecular reactions. Highly degraded DNA from wood is also difficult to be amplified for large amplicons in standard barcoding regions. Shorter DNA amplicons have to be developed for species identification using DNA mini-barcoding^21–24^. However, shorter amplicons provide less sequence information and limit their discriminatory power. This presents difficulty in species-level identification for the law enforcement as well as for research and conservation of rosewood and agarwood. On the other hand, although a number of dry wood DNA extraction protocols have been published, those protocols were mostly performed on only one wood genus^25–28^. They were rarely performed on processed rosewood and agarwood products to species-level identification.

In this study, we evaluated and compared three modified DNA extraction protocols in extracting amplifiable DNA from different parts of authentic rosewood (*Dalbergia* and *Pterocarpus*) and agarwood (*Aquilaria*) samples, and applied the best DNA extraction protocol on processed commercial wood products which were labelled as rosewood and agarwood. We also demonstrated the use of DNA mini-barcoding with multi-loci combination with reference library for identifying the species of authentic wood samples and commercial wood products. The outcome of this study provides a suitable identification method for rosewood and agarwood timber logs and processed products, facilitating the forensic timber identification and the enforcement of CITES control and benefiting conservation and forest protection.

## Materials and methods

### Sources of samples

In this study, 12 authentic wood samples of *Aquilaria*, *Dalbergia* and *Pterocarpus* were from the collection of Shiu-Ying Hu Herbarium and donations of Agriculture, Fisheries and Conservation Department of the Hong Kong Special Administrative Region Government (AFCD of HKSAR Government). For commercial wood products, four samples were donated by AFCD of HKSAR Government and six samples were purchased from local retail stores, either ordered online or bought from physical stores. Leaf and twig samples were also collected as positive controls during amplification and for generating reliable reference barcode sequences. These samples were collected from Hong Kong by the staff members of Shiu-Ying Hu Herbarium of The Chinese University of Hong Kong with deposited voucher specimens. The collections are permitted under the *Permission to Make Field Collection for Research Purpose* issued by AFCD of HKSAR Government. Two samples, namely KFBG_7521 and KFBG_9203, were sampled from the living individuals cultivated in Kadoorie Farm & Botanic Garden in Hong Kong on 25^th^ September, 2019. All collections are permitted and legal in Hong Kong. Details of collected authentic wood samples, commercial wood products and reference leaf and twig samples are listed in Tables 1, 2 and Supplementary Table S1, respectively. Figure S1 shows the photos of the authentic wood samples, with different sampled parts annotated. Photos of the commercial wood products are shown in Fig. S2.

**Table 1:** List of authentic wood samples collected.

Sample code	Route of collection	Claimed genus	Type of sample	Multi-loci combination used for identification	Species of origin identified through multi-loci combination
T5196	Fallen wood log originally grown in Hong Kong, China	*Aquilaria*	Sapwood (T5196S)	ITS2 + *trnL-trnF*	*Aquilaria sinensis*
			Outer heartwood (T5196O)	ITS2 + *trnL-trnF*	*Aquilaria sinensis*
			Inner heartwood (T5196I)	ITS2 + *trnL-trnF*	*Aquilaria sinensis*
T4960	Donated by AFCD, HKSAR	*Aquilaria*	Unknown	ITS2 + *trnL-trnF*	*Aquilaria beccariana*
T5149	Donated by AFCD, HKSAR	*Aquilaria*	Sapwood	ITS2 + *trnL-trnF*	*Aquilaria sinensis*
T5154	Donated by AFCD, HKSAR	*Aquilaria*	Unknown	ITS2 + *trnL-trnF*	*Aquilaria beccariana*
T5155	Donated by AFCD, HKSAR	*Aquilaria*	Unknown	ITS2 + *trnL-trnF*	*Aquilaria beccariana*
T4961	Donated by AFCD, HKSAR	*Dalbergia*	Sapwood (T4961S)	ITS2 + *matK*	*Dalbergia stevensonii*
			Heartwood (T4961H)	ITS2 + *matK*	*Dalbergia stevensonii*
T4966	Donated by AFCD, HKSAR	*Dalbergia*	Sapwood (T4966S)	ITS2 + *matK*	*Dalbergia oliveri*
			Outer heartwood (T4966O)	ITS2 + *matK*	*Dalbergia oliveri*
			Inner heartwood (T4966I)	ITS2 + *matK*	*Dalbergia oliveri*
T5152	Donated by AFCD, HKSAR	*Dalbergia*	Sapwood (T5152S)	ITS2 + *matK*	*Dalbergia oliveri*
			Outer heartwood (T5152O)	ITS2 + *matK*	*Dalbergia oliveri*
			Inner heartwood (T5152I)	ITS2 + *matK*	*Dalbergia oliveri*
T5153	Donated by AFCD, HKSAR	*Dalbergia*	Heartwood	ITS2 + *matK*	*Dalbergia stevensonii*
T4964	Donated by AFCD, HKSAR	*Pterocarpus*	Outer heartwood (T4964O)	ITS2 + *matK*	*Pterocarpus santalinus*
			Inner heartwood (T4964I)	ITS2 + *matK*	*Pterocarpus santalinus*
T4965	Donated by AFCD, HKSAR	*Pterocarpus*	Outer heartwood (T4965O)	ITS2 + *matK*	*Pterocarpus santalinus*
			Inner heartwood (T4965I)	ITS2 + *matK*	*Pterocarpus santalinus*
T5197	Fallen wood log originally grown in Hong Kong, China	*Pterocarpus*	Sapwood (T5197S)	ITS2 + *matK*	*Pterocarpus santalinus*
			Outer heartwood (T5197O)	ITS2 + *matK*	*Pterocarpus santalinus*
			Inner heartwood (T5197I)	ITS2 + *matK*	*Pterocarpus santalinus*

**Table 2:** List of commercial wood products collected.

Sample code	Sample form	Route of collection	Place of collection	Claimed place of origin	Claimed species of origin	Species referred to Chinese common name	Product trade name/ Description	Multi-loci combination used for identification	Species of origin identified through multi-loci combination	Discriminatory level	Accuracy
T4959	Wood chips	Donated by AFCD, HKSAR	Hong Kong, China	Unknown	*Aquilaria* sp.	N/A	Agarwood chip	ITS2 + *trnL-trnF*	*Aquilaria beccariana*	Species-level	Correct
T5300	Powder	Purchased from physical store	Xiamen, China	Unknown	*Aquilaria* sp.	N/A	*Aquilaria* powder (沉香粉)	ITS2 + *matK*	*Aquilaria crassna*	Species-level	Correct
T5339	Incense stick	Purchased from online store	Hong Kong, China	Vietnam	*Aquilaria* sp.	N/A	*Aquilaria* stick (沉香)	ITS2 + *matK*	*Aquilaria crassna*	Species-level	Correct
T5204	Beads from bracelet	Purchased from physical store	Yunnan, China	Unknown	*Dalbergia nigra*	*Dalbergia nigra*^1^	*Dalbergia nigra* (黑檀)	ITS2 + *matK*	*Dalbergia melanoxylon*	Species-level	Incorrect
T5348	Wooden disc	Purchased from online store	Hong Kong, China	India	*Dalbergia sissoo*	*Dalbergia sissoo*^2^	*Dalbergia sissoo* round disc (印度黃檀)	ITS2 + *trnL*	*Dalbergia sissoo*	Species-level	Correct
T4962	Beads from necklace	Donated by AFCD, HKSAR	Hong Kong, China	Unknown	*Pterocarpus santalinus*	N/A	Red sandalwood beads	ITS2 + *matK*	*Pterocarpus santalinus*	Species-level	Correct
T4963	Wood block from furniture part	Donated by AFCD, HKSAR	Hong Kong, China	Unknown	*Pterocarpus santalinus*	N/A	Red sandalwood furniture parts	ITS2 + *matK*	*Pterocarpus santalinus*	Species-level	Correct
T5150	Wood block	Donated by AFCD, HKSAR	Hong Kong, China	Unknown	*Pterocarpus santalinus*	N/A	Red sandalwood part	ITS2 + *matK*	*Pterocarpus santalinus*	Species-level	Correct
T5338	Wood piece	Purchased from online store	Hong Kong, China	Hong Kong, China	Unknown	*Pterocarpus* spp.^3^	Rosewood (*Pterocarpus*) wood piece (花梨木)	ITS2 + *matK*	*Pterocarpus indicus*	Species-level	Correct
T5205	Beads from bracelet	Purchased from physical store	Yunnan, China	Unknown	Unknown	*Dalbergi*a spp*.*^3^	Rosewood (*Dalbergia*) bracelet (酸枝木)	ITS2 + *matK*	*Pterocarpus indicus*	Species-level	Incorrect

^1^Protection of Endangered Species of Animals and Plants Ordinance, Cap 586 (2018).

^2^Chiayi Botanical Garden. Chungpu Research Center, Forestry Research lnstitute, Council of Agriculture of Taiwan. Retrieved from https://cptfri.tfri.gov.tw/ on 01 March, 2021.

^3^The National Standards of the Republic of China (GBT18107-2017, “Hongmu”). General Administration of Quality Supervision, Inspection & Quarantine of the People's Republic of China (AQSIQ), Standardization Administration of the People's Republic of China (SAC). Retrieved from http://www.gb688.cn/bzgk/gb/newGbInfo?hcno=6E961C6DB78254EF883B5053D08BFA3B on 01 March, 2021.

### Genomic DNA extraction

For leaf and twig samples used as reference, DNA of these samples were extracted using BioMed Plant Genome DNA Extraction Kit (Biomed, Beijing, China) following manufacturer’s instructions. Small wood pieces were first sampled from both authentic and commercial wood products using electric drill or scalpel blades and further disrupted into fine powder using Precellys Evolution Tissue Homogenizer (Bertin Technologies, Montigny-le-Bretonneux, France). For authentic wood samples T5196, T4966, T5155, T5152 and T5197, small wood pieces were taken from three parts namely sapwood, outer heartwood and inner heartwood following the practice of Rachmayanti^27^ and Jiao^13^. Inner heartwood was sampled about 2 cm diameter from pith, while outer heartwood was defined as the transition zone between sapwood and inner heartwood. For authentic wood samples T4964 and T4965, since sapwood is absent, only the outer and inner heartwood were sampled. Because authentic wood sample T4961 is an incomplete wood block lacking the inner heartwood, small wood pieces of sapwood and heartwood were taken, without further differentiating the heartwood into inner and outer part.

The quality, quantity and amplifiability of DNA extracted using three different genomic DNA extraction methods, including SDS-based method, QIAGEN method and another silica-column-based method using guanidine thiocyanate (GuSCN)-containing binding buffer developed by The Canadian Centre for DNA Barcoding (CCDB method), were compared. For fair comparison, the amount of wood powder and the amount of water/elution buffer used to elute DNA were kept the same. The same silica columns, QIAquick Spin Columns (Qiagen, Hilden, Germany), were used in all three methods. After evaluating the results of DNA extracted from the three DNA extraction methods, DNA of commercial wood products were extracted using the CCDB method.

#### SDS-based method

The extraction protocol was modified from Little^29^. A total of 0.3 g wood powder were mixed with 0.1 g of polyvinylpolypyrrolidone (PVPP) and incubated with 2 mL of extraction buffer (8 mM NaCl, 16 mM sucrose, 5.8 mM EDTA, 0.5% (w/v) sodium dodecyl sulphate, 12.4 mM Tris (pH 7.4)) and 20 µL of proteinase K (20 mg/mL) for 18 h at 42 °C. After incubation, 400 µL of 3 M potassium acetate (pH 4.7) was added followed by incubation at 0 °C for 10 min. After incubation, mixtures were centrifuged at 15,000 rpm for 5 min. Clear supernatant was collected and mixed with 1.5 × volume of 2 M guanidine hydrochloride in 95% (v/v) ethanol. Mixtures were applied to QIAquick Spin Columns followed by centrifugation at 5000 rpm for 5 min. After all mixtures were applied, sample was washed twice with 500 µL of washing buffer (50% (v/v) ethanol, 10 mM Tris (pH 7.4), 0.5 M EDTA, 50 mM NaCl) with centrifugation at 5000 rpm at 5 min. Ethanol residue was removed by centrifugation at 15,000 rpm for 5 min. Genomic DNA were eluted with 50 µL of pre-warmed molecular biology grade water with centrifugation at 15,000 rpm for 5 min after incubation at room temperature for 2–3 min. Time consumed in this method was approximately within 20 h for 24 samples and each sample costed USD 6.4.

#### QIAGEN method

DNeasy Plant Pro Kit (Qiagen, Hilden, Germany) was used according to manufacturer’s instructions with modification. A total of 0.3 g wood powder was first mixed with 0.1 g of PVPP and incubated with 1.8 mL of CD1 solution and 200 µL of PS solution for 1 h at 65 °C. After incubation, mixtures were centrifuged at 15,000 rpm for 20 min. Clear supernatant was collected and mixed with same volume of solution CD2 followed by centrifugation at 15,000 rpm for 20 min. Clear supernatant was collected and mixed with 1000 µL of APP solution. Mixtures were applied to QIAquick Spin Columns followed by centrifugation at 5000 rpm for 5 min. After all mixtures were applied, column was washed with 650 µL of AW1 solution followed by centrifugation at 5000 rpm at 5 min and 650 µL of AW2 solution followed by centrifugation at 5000 rpm at 5 min. Ethanol residue was removed by centrifugation at 15,000 rpm for 5 min. Genomic DNA were eluted with 50 µL of pre-warmed EB solution with centrifugation at 15,000 rpm for 5 min. Time consumed in this method was approximately within 3.5 h for 24 samples and each sample costed USD 17.

#### CCDB method

Genomic DNA was extracted using extraction protocol modified from Ivanova^30^. A total of 0.3 g sample powder was mixed with 0.1 g of PVPP and mixed with 2 mL of lysis buffer (700 mM GuSCN, 30 mM EDTA (pH 8.0), 30 mM Tris–HCl (pH 8.0), 0.5% Triton X-100, 5% Tween-20), 200 µL of proteinase K (20 mg/mL) and 50 µL of α-amylase. The mixtures were incubated at 56 °C for 30 min followed by an incubation at 65 °C for 1 h. After incubation, mixtures were centrifuged at 15,000 rpm for 10 min and clear supernatant was collected and mixed with equal volume of extraction binding buffer (6 M GuSCN, 20 mM EDTA (pH 8.0), 10 mM Tris–HCl (pH 6.4), 4% Triton X-100). Mixtures were applied to QIAquick Spin Columns followed by centrifugation at 5000 rpm for 5 min. After all mixtures were applied, sample was washed with 180 µL of extraction wash buffer (60% ethanol, 50 mM NaCl, 10 mM Tris–HCl (pH 7.4), 50 mM EDTA (pH 8.0)) with centrifugation at 5000 rpm for 5 min. After that, sample was washed twice with 650 µL of extraction wash buffer. Ethanol residue was removed by centrifugation at 15,000 rpm for 5 min. Genomic DNA were eluted with 50 µL of pre-warmed water with centrifugation at 15,000 rpm for 5 min after incubation at room temperature for 2–3 min. Time consumed in this method was approximately within 4 h for 24 samples and each sample costed USD 13.5.

### DNA quantity and quality analysis

For comparison of DNA extracted using the three genomic DNA extraction methods, the quality and quantity of the DNA extracted were evaluated by a NanoDrop Lite spectrophotometer (Thermo Scientific, Massachusetts, USA).

### PCR amplification, agarose gel electrophoresis and DNA sequencing

For each genus, different DNA mini-barcode loci were selected for amplification and sequencing based on their species discrimination ability evaluated by previous research. For *Aquilaria*, DNA barcode loci ITS2, *matK* and *trnL*-*trnF* were selected^20^. For *Dalberiga*, DNA barcode loci ITS2, *matK*, *trnH*-*psbA* and *trnL* were selected^18^ and for *Pterocarpus*, DNA barcode loci ITS2, *matK*, *rbcL* and *ndhF*-*rpl32* were selected^19^. DNA extracted were amplified using the GoTaq G2 Flexi DNA Polymerase (Promega, Wisconsin, USA). Each 15-μL PCR contained 10 ng template DNA, 3 μL 5X Colorless GoTaq Flexi Buffer, 2.4 μL MgCl_2_ (25 mmol/L), 0.3 μL dNTP mixture (10 mmol/L each), 0.75 μL forward primer (10 μmol/L), 0.75 μL reverse primer (10 μmol/L) and 0.125 μL GoTaq polymerase (5 U/μL). Sequences of primers and the corresponding reaction conditions are listed in Supplementary Table S2. PCR products were mixed with 6X loading dye in a ratio of 5:2 and visualized in 2% agarose gel and sizes of fragments were compared with GeneRuler 100 bp DNA Ladder. After evaluating the result of DNA extracted from the three DNA extraction methods, PCR products with successful amplification of DNA extracted with the CCDB method were purified with Biomed gel extraction kit (Biomed, Beijing, China). Sanger sequencing was performed by Tech Dragon Ltd., Hong Kong.

### Additional sequences

In addition to sequences generated from reference leaf and twig samples, sequences of *Aquilaria* (ITS2, *matK* and *trnL*-*trnF*), *Dalberiga* (ITS2, *matK*, *trnH*-*psbA* and *trnL*) and *Pterocarpus* (ITS2, *matK*, *rbcL* and *ndhF*-*rpl32*) were downloaded from GenBank for data analysis (Supplementary Tables S3-S5).

### Data analysis

Raw sequences generated from reference leaf and twig samples in this study were assembled and aligned followed by a manual adjustment using BioEdit^31^, saved in FASTA format and deposited to GenBank. Reference DNA barcode libraries for each genus (*Aquilaria, Dalbergia* and *Pterocarpus*) were developed from sequences obtained from leaf and twig samples and sequences obtained from GenBank for species identification of authentic wood samples and commercial products based on single DNA barcodes and multi-loci combinations through phylogenetic tree-based analysis. Phylogenetic trees were constructed based on the aligned sequences from single loci or concatenated loci using neighbour-joining (NJ) method using the Kimura 2-Parameter (K2P) model^32^ with bootstrap re-sampling (n = 1000) and pairwise deletion using MEGA X^33^.

### Plant collection statement

All plant samples in this study were collected in Hong Kong and the experiments were performed locally. The authors carefully checked that proper sample collection permits were in place at the time of collection. The authors declare that all the experimental research and field sampling of plant material comply with institutional, local, national and international guidelines and legislation.

## Results

### Comparison of three genomic DNA extraction methods

The quality and quantity of DNA extracted from wood samples of all three genera are shown in Table 3. For the quantity of extracted DNA, the yield of DNA obtained using SDS-based method was the highest, in a range of 5.1–971.0 ng/μL, followed by CCDB method, in a range of 5.8–270.5 ng/μL, and QIAGEN method, in a range of 1.1–12.1 ng/μL. For wood samples which contain both sapwood and heartwood, higher DNA yield were obtained from sapwood than heartwood for all three extraction methods. The quality of extracted DNA of each extraction method varies across the authentic wood samples (Table 3).

**Table 3:** Quality and quantity of DNA extracted from authentic wood samples using three genomic DNA extraction methods.

Sample code	Claimed genus	Type of sample	SDS-based method		QIAGEN method		CCDB method	
			DNA concentration (ng/μL)	Purity (OD 260/280 ratio)	DNA concentration (ng/μL)	Purity (OD 260/280 ratio)	DNA concentration (ng/μL)	Purity (OD 260/280 ratio)
T5196	*Aquilaria*	Sapwood (T5196S)	491.1	2.00	4.5	2.39	108.4	1.86
		Outer heartwood (T5196O)	220.3	1.93	1.9	2.17	8.5	1.82
		Inner heartwood (T5196I)	209.7	1.95	1.9	1.95	19.5	1.84
T4960	*Aquilaria*	Unknown	236.2	2.62	4.7	3.52	12.4	1.96
T5149	*Aquilaria*	Sapwood	580.7	1.32	12.1	1.72	270.5	1.79
T5154	*Aquilaria*	Unknown	177.5	2.50	1.3	2.11	15.2	2.05
T5155	*Aquilaria*	Unknown	154.9	1.46	3.3	1.72	21.6	1.71
T4961	*Dalbergia*	Sapwood (T4961S)	118.3	1.46	10.2	1.70	68.7	1.69
		Heartwood (T4961H)	53.7	1.77	2.3	1.82	25.9	1.45
T4966	*Dalbergia*	Sapwood (T4966S)	960.0	1.07	6.8	1.45	61.7	1.32
		Outer heartwood (T4966O)	47.5	1.70	3.4	1.54	15.6	1.31
		Inner heartwood (T4966I)	64.6	1.68	3.2	1.57	12.7	2.20
T5152	*Dalbergia*	Sapwood (T5152S)	971.0	1.08	4.8	1.31	41.9	1.34
		Outer heartwood (T5152O)	78.2	1.50	1.7	1.46	14.6	1.44
		Inner heartwood (T5152I)	47.6	1.72	2.4	1.59	20.8	1.47
T5153	*Dalbergia*	Heartwood	7.6	1.68	1.6	1.35	7.9	1.19
T4964	*Pterocarpus*	Outer heartwood (T4964O)	11.8	0.66	1.1	1.53	6.5	1.22
		Inner heartwood (T4964I)	6.4	1.21	2.1	1.53	5.8	1.35
T4965	*Pterocarpus*	Outer heartwood (T4965O)	5.1	1.19	2.6	1.48	9.5	1.29
		Inner heartwood (T4965I)	25.5	1.27	3.4	1.43	10.2	1.50
T5197	*Pterocarpus*	Sapwood (T5197S)	429.4	1.65	1.5	1.35	24.5	1.64
		Outer heartwood (T5197O)	413.5	1.20	1.2	1.34	21.3	1.23
		Inner heartwood (T5197I)	368.5	1.34	1.8	1.51	25.2	1.20

**Figure 1:**
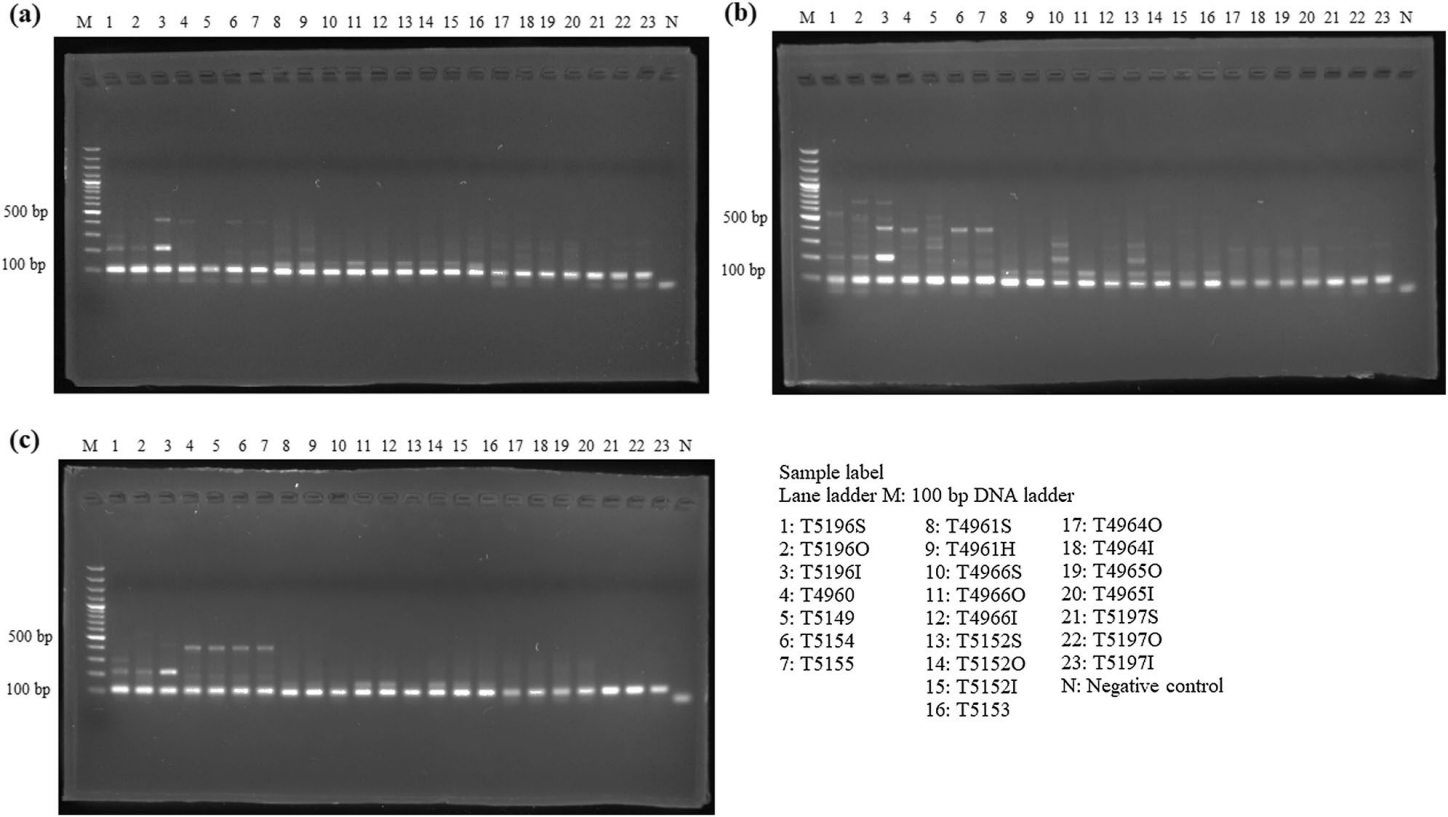
Gel electrophoresis of PCR amplification results of all authentic wood samples using different extraction methods, (**a**) SDS-based method, (**b**) QIAGEN method, and (**c**) CCDB method, with internal control primer set *trnL* gh.

**Figure 2:**
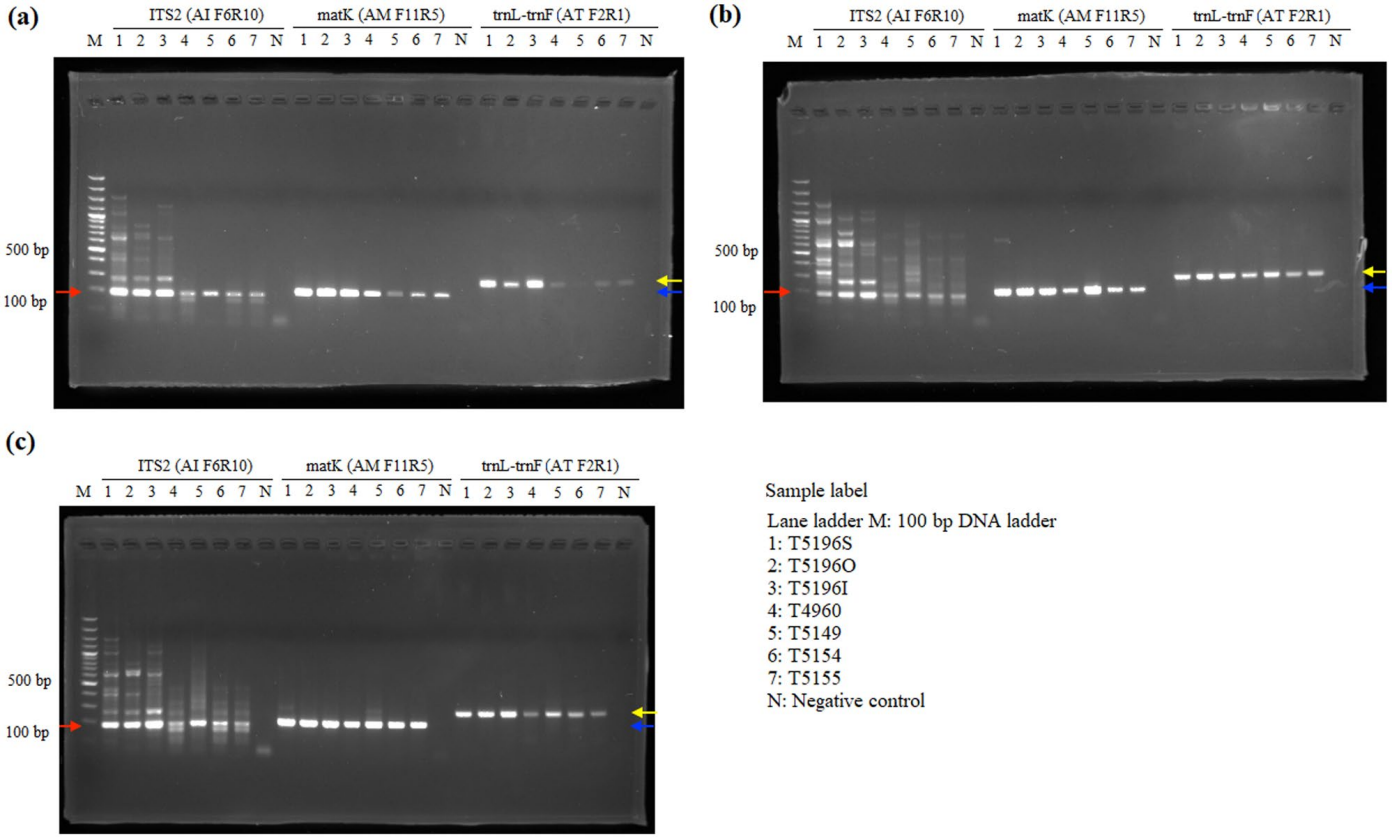
Gel electrophoresis of PCR amplification results of *Aquilaria* authentic wood samples using different extraction methods, (**a**) SDS-based method, (**b**) QIAGEN method, and (**c**) CCDB method. Red arrows indicate the amplicons of ITS2 (181 bp). Blue arrows indicate the amplicons of *matK* (209 bp). Yellow arrows indicate the amplicons of *trnL*-*trnF* (280 bp).

**Figure 3:**
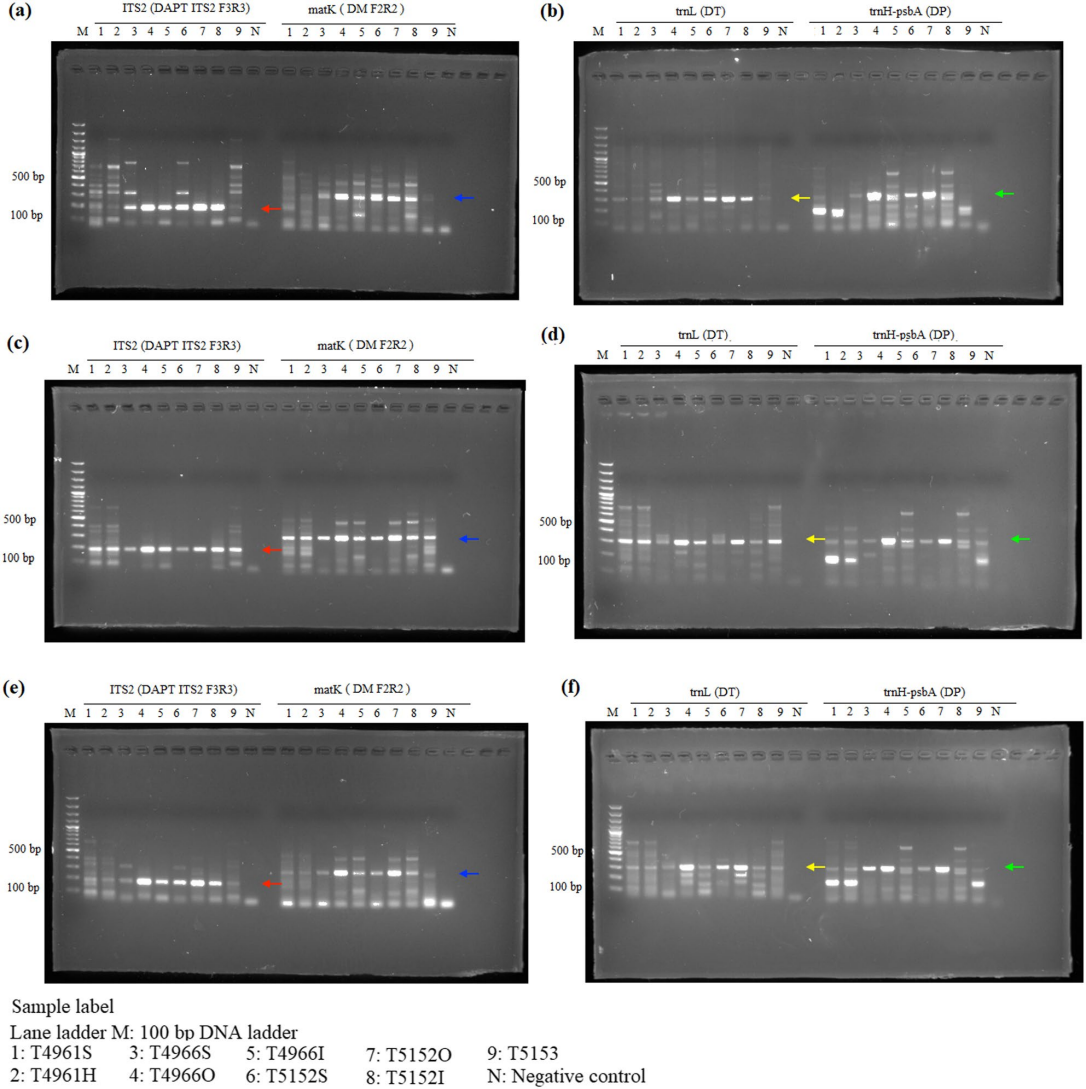
Gel electrophoresis of PCR amplification results of *Dalbergia* authentic wood samples using different extraction methods, (**a**,**b**) SDS-based method, (**c**,**d**) QIAGEN method, and (**e**,**f**) CCDB method. Red arrows indicate the amplicons of ITS2 (180 bp). Blue arrows indicate the amplicons of *matK* (300 bp). Yellow arrows indicate the amplicons of *trnL* region (314 bp). Green arrows indicate the amplicons of *trnH*-*psbA* (334 bp).

**Figure 4:**
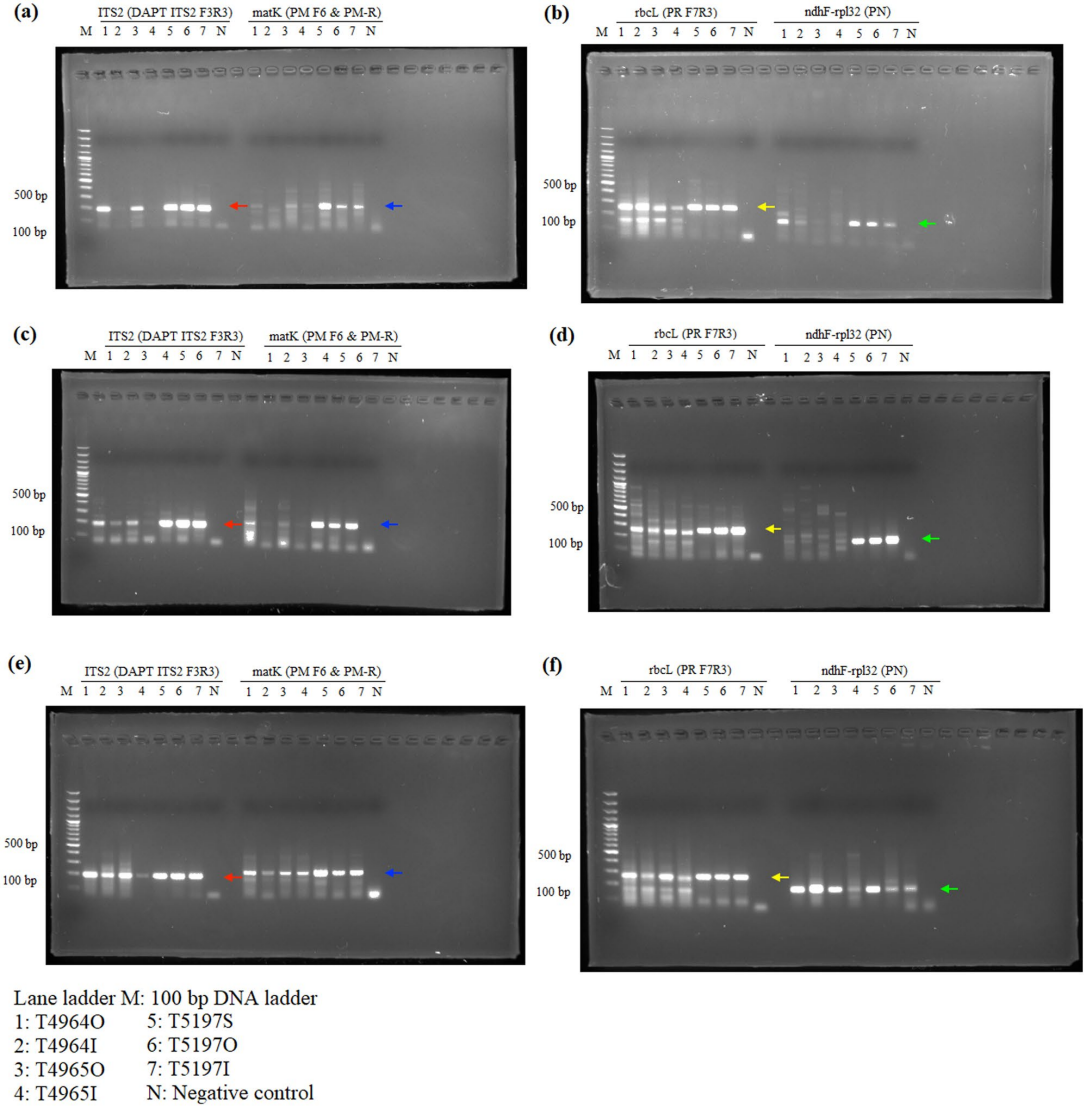
Gel electrophoresis of PCR amplification results of *Pterocarpus* authentic wood samples using different extraction methods, (**a**,**b**) SDS-based method, (**c**,**d**) QIAGEN method, and (**e**,**f**) CCDB method. Red arrows indicate the amplicons of ITS2 (180 bp). Blue arrows indicate the amplicons of *matK* (200 bp). Yellow arrows indicate the amplicons of *rbcL* (250 bp). Green arrows indicate the amplicons of ndhF-rpl32 (167–173 bp).

The P6-loop of *trnL*(UAA) was selected as an internal control for showing amplifiability of DNA extracts, because of the availability of universal primers that produce short amplicons. For the amplifiability of extracted DNA, DNA samples extracted from all three extraction methods showed positive results in internal control and no amplification was found in the negative control, showing amplifiable DNA were extracted from all three methods (Fig. 1). For *Aquilaria* wood samples, all three target regions, ITS2, *matK* and *trnL*-*trnF*, were amplified with expected amplicon sizes from DNA samples extracted using all three methods (Fig. 2). However, for *trnL*-*trnF* region, weaker band was found from DNA samples extracted using the SDS-based method. For *Dalbergia* wood samples, positive results with expected amplicon sizes were found in amplification of ITS2, *matK*, *trnH*-*psbA* and *trnL* regions with samples extracted using QIAGEN and CCDB methods (Fig. 3). For wood samples T4966 and T5152, which contain both sapwood and heartwood sections, stronger amplification was found with DNA extracted from the sapwood section than those from heartwood section. On the other hand, for the SDS-based method, amplification of *matK* and *trnH*-*psbA* regions from samples T4961 and T5153 failed to produce target amplicons of the right sizes. For *Pterocarpus* wood sample, positive results with the expected amplicon sizes were found from all samples extracted using the CCDB method in ITS2, *matK*, *rbcL* and *ndhF*-*rpl32* regions (Fig. 4). On the other hand, for DNA extracted from sample T4964 and T4965 using QIAGEN method and SDS-based method, weak amplification in non-target sizes were found in amplification targeting *matK* and *ndhF*-*rpl32* regions. Negative result was also found with amplification of targeting ITS2 region with DNA extracted from the inner heartwood section of sample T4964 and T4965 using the SDS-based method. Overall, better amplification results were found in DNA extracts using CCDB method.

### Phylogenetic tree analysis for authentic wood samples

Species of wood samples of all three genera could be successfully discriminated using neighbour-joining trees with multi-loci combination constructed based on reference DNA barcode libraries (Table 4 and Supplementary Figs. S3-S39). Single DNA barcode is not sufficient to discriminate the species of all authentic wood samples. For some of the wood samples, neighbour-joining trees constructed with different single DNA barcode regions would lead to different identification results. Comparing to other single DNA barcode region, ITS2 generally resulted in the highest species discrimination success rate for all three genera while *matK*, *rbcL* and *ndhF*-*rpl32* for *Pterocarpus* were not able to discriminate any authentic wood samples to species-level. On the other hand, multi-loci combination generally resulted in a higher species discrimination success rate. Highest discrimination success rate (100%) was obtained with combination of *trnL*-*trnF* + ITS2 and *matK* + *trnL*-*trnF* + ITS2 for *Aquilaria*, all multi-loci combinations except for *matK* + *trnL* for *Dalbergia*, and combinations of ITS2 + *matK*, ITS2 + *matK* + *rbcL*, ITS2 + *matK* + *ndhF*-*rpl32* and ITS2 + *matK* + *rbcL* + *ndhF*-*rpl32* for *Pterocarpus*.

**Table 4:** Species discrimination success rate of *Aquilaria, Dalbergia* and *Pterocarpus* authentic wood sample using the neighbor-joining analysis based on single DNA regions and multi-loci combinations.

Genus	DNA barcode region and combination	Discrimination success rate	Number of monophyletic-species clade
*Aquilaria*	ITS2	40% (2/5)	3
	*matK*	40% (2/5)	1
	*trnL-trnF*	40% (2/5)	1
	ITS2 + *matK*	80% (4/5)	3
	ITS2 + *trnL-trnF*	100% (5/5)	5
	*matK* + *trnL-trnF*	80% (4/5)	1
	ITS2 + *matK* + *trnL-trnF*	100% (5/5)	4
*Dalbergia*	ITS2	100% (4/4)	6
	*matK*	50% (2/4)	7
	*trnL*	50% (2/4)	9
	*trnH-psbA*	100% (4/4)	10
	ITS2 + *matK*	100% (4/4)	12
	ITS2 + *trnH-psbA*	100% (4/4)	7
	ITS2 + *trnL*	100% (4/4)	5
	*matK* + *trnH-psbA*	100% (4/4)	9
	*matK* + *trnL*	50% (2/4)	7
	*psbA-trnH* + *trnL*	100% (4/4)	7
	ITS2 + *matK* + *trnH-psbA*	100% (4/4)	10
	ITS2 + *matK* + *trnL*	100% (4/4)	6
	ITS2 + *psbA-trnH* + *trnL*	100% (4/4)	6
	*matK* + *psbA-trnH* + *trnL*	100% (4/4)	6
	ITS2 + *matK* + *trnH-psbA* + *trnL*	100% (4/4)	5
*Pterocarpus*	ITS2	66.67% (2/3)	3
	*matK*	0% (0/3)	0
	*rbcL*	0% (0/3)	2
	*ndhF-rpl32*	0% (0/3)	0
	ITS2 + *matK*	100% (3/3)	1
	ITS2 + *rbcL*	66.67% (2/3)	2
	ITS2 + *ndhF-rpl32*	66.67% (2/3)	1
	*matK* + *rbcL*	66.67% (2/3)	2
	*matK* + *ndhF-rpl32*	66.67% (2/3)	1
	*rbcL* + *ndhF-rpl32*	66.67% (2/3)	2
	ITS2 + *matK* + *rbcL*	100% (3/3)	2
	ITS2 + *matK* + *ndhF-rpl32*	100% (3/3)	2
	ITS2 + *rbcL* + *ndhF-rpl32*	66.67% (2/3)	2
	*matK* + *rbcL* + *ndhF-rpl32*	66.67% (2/3)	2
	ITS2 + *matK* + *rbcL* + *ndhF-rpl32*	100% (3/3)	2

To study the species-level resolution, the number of species identifiable by each locus/multi-loci combination, i.e. forming monophyletic-species clade in the NJ phylogenetic tree, are also indicated in Table 4. Taking the discrimination success rate and the number of species with monophyletic clades formed into account, DNA barcode region ITS2 + *trnL*-*trnF* can provide the highest rate of species resolution (100%) and creating 5 monophyletic clades in *Aquilaria*. ITS2 + *matK* performed the best in identifying species of *Dalbergia* with the highest discrimination rate (100%) and 12 monophyletic clades. For the genus *Pterocarpus*, among 15 DNA barcode regions and combinations, ITS2 + *matK* + *rbcL*, ITS2 + *matK* + *ndhF*-*rpl32* and ITS2 + *matK* + *rbcL* + *ndhF*-*rpl32* worked well with 100% successful identification rate and formed 2 monophyletic clades respectively.

### Species identification of commercial wood products

After evaluating the results obtained from authentic wood samples, CCDB method was also applied on the 10 commercial wood products. Internal control was successfully amplified from all 10 commercial products. For each product, positive amplification was found in at least two DNA barcode regions (Fig. 5). Using the neighbour-joining trees constructed based on the reference DNA barcode libraries, species of commercial wood products were identified (Table 2, Fig. 6 and Supplementary Figs. S40-S76). Based on the list of CITES Appendix and the GB/T 18107-2017, all 10 commercial products were identified to species-level. Our identification results showed that 80% of the commercial products were labelled correctly.

**Figure 5:**
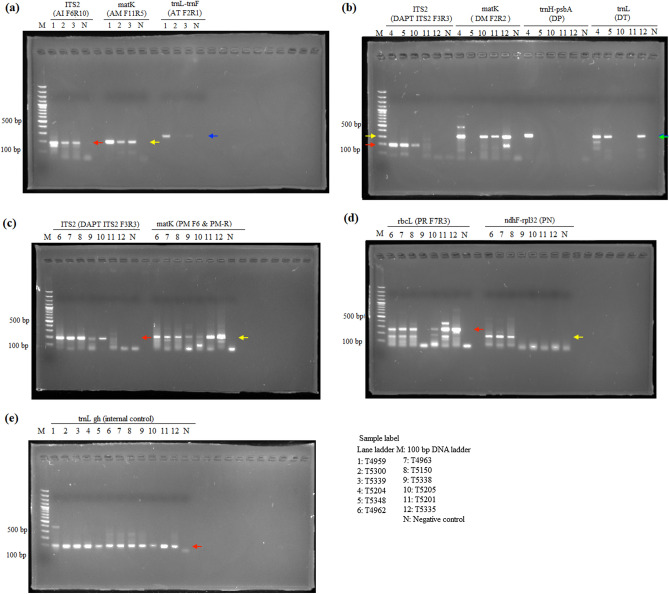
Gel electrophoresis of PCR amplification results of commercial wood product using CCDB method with (**a**) agarwood samples (red arrow—ITS2 at 181 bp, yellow arrow—*matK* at 209 bp, blue arrow—*trnL*-*trnF* at 280 bp); (**b**) rosewood samples (red arrow—ITS2 at 180 bp, yellow arrow—*matK* at 300 bp, blue arrow—*trnH*-*psbA* at 334 bp, green arrow—*trnL* at 314 bp); (**c**) rosewood samples (red arrow—ITS2 at 180 bp, yellow arrow—*matK* at 200 bp); (**d**) rosewood samples (red arrow—*rbcL* at 259 bp, yellow arrow—*ndhF*-*rpl32* at 167–173 bp); (**e**) all 10 commercial samples amplified with internal control primers (red arrow—internal control amplicon).

**Figure 6:**
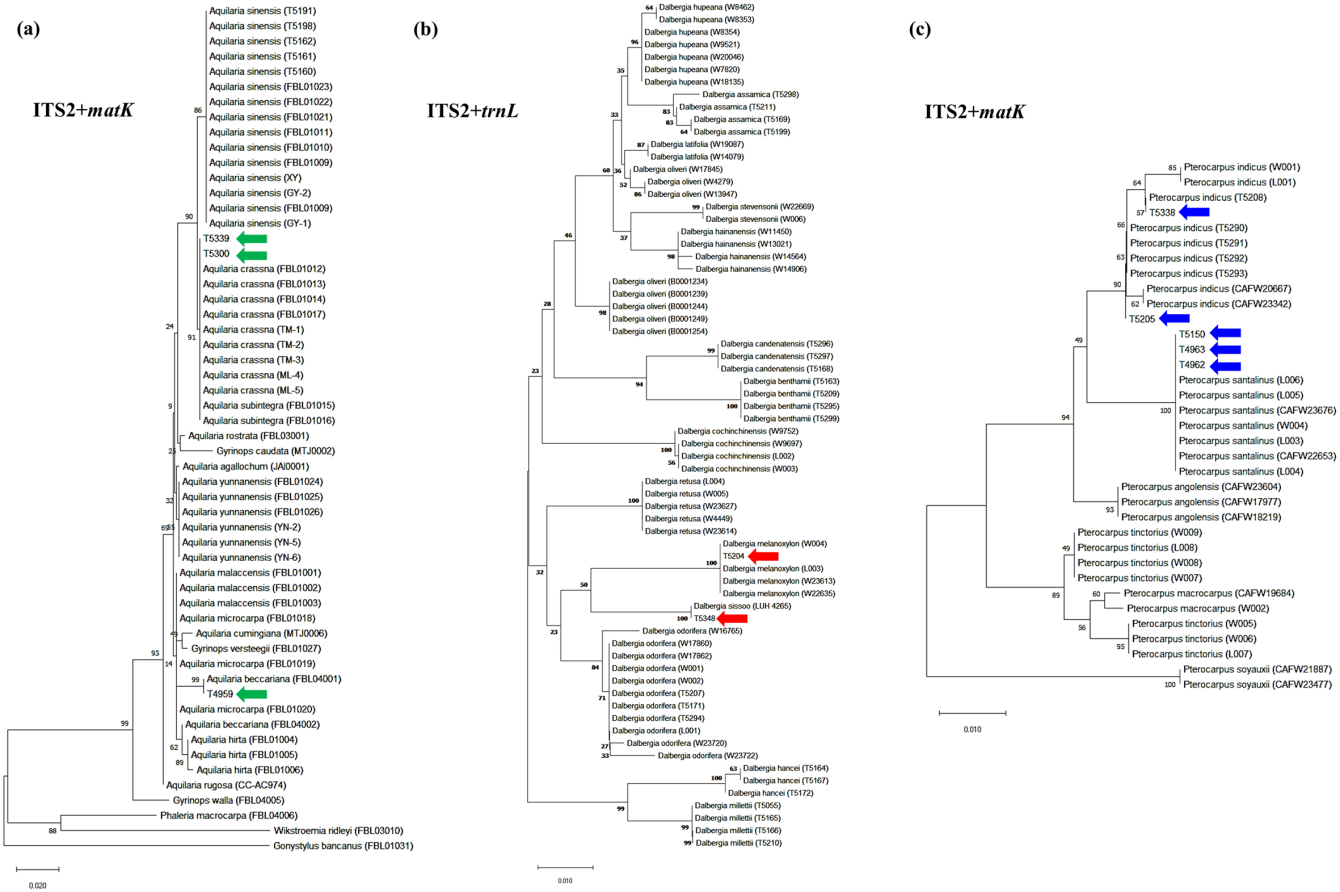
Neighbour-joining trees constructed based on the reference DNA barcode libraries identifying species of commercial wood products using multi-loci combination (**a**) ITS2 + *matK* for *Aquilaria* commercial wood products indicated by green arrows, (**b**) ITS2 + *trnL* for *Dalbergia* commercial wood products indicated by red arrows and (**c**) ITS2 + *matK* for *Pterocarpus* commercial wood products indicated by blue arrows.

## Discussion

DNA barcoding has been shown to be highly effective for the identification of wood species. However, DNA extraction from dried, aged authentic wood samples and processed commercial wood products have not been easy due to the highly degraded DNA in wood sample and the presence of high quantity of polysaccharides and phenolic compounds, which can be difficult to be separated from DNA and inhibit PCR amplification^28,34^. In this work, we have successfully compared three genomic DNA extraction methods on authentic wood samples of *Aquilaria*, *Dalbergia* and *Pterocarpus* and demonstrated species identification on both authentic wood samples and commercial wood products using neighbour-joining tree and multi-loci combination.

Among all three extraction methods, extracted DNA from CCDB method resulted the best in purity and amplifiability. Only DNA extracted from CCDB method were able to achieve 100% amplification success rate with all samples and their corresponding target DNA barcodes. Although the cost of CCDB method was twice of the cost of SDS-based method, the cost difference is mainly contributed by the use of guanidine thiocyanate (GuSCN). GuSCN in lysis buffer along with proteinase K and α-amylase successfully helps denaturing protein and catalyses the breakdown of polysaccharides in wood sample containing lignin and cellulose in the lysis step. High content of GuSCN in extraction binding buffer also enhances the binding of DNA with silica on the binding column^35^, reducing the DNA loss when flowing through the column and yielding DNA with good amplifiability. Reduction of DNA loss during extraction is fundamental especially for aged wood samples with highly degraded DNA and in limited amount. On the other hand, although DNA yielded from SDS-based method resulted in the highest quantity, the quality and amplification success rate were low. This was especially so for *Dalbergia* and *Pterocarpus* heartwood, which contain high content of retusapurpurins A and B and pterocarpan, respectively^36,37^. This shows that the SDS-based method may be less effective in separating phenolic compounds from DNA and less suitable for extracting DNA from heartwood samples. For QIAGEN method, although it required the shortest time for extraction, DNA yielded by QIAGEN method had a low quantity and the amplification success rate was lower than that of CCDB method on *Pterocarpus* heartwood sample. Furthermore, since the quantity of DNA yielded were generally under 10 ng/μL, more DNA extracts must be used to reach 10 ng of template DNA for each PCR amplification, which highly increased the cost for the identification of a sample. Overall, DNA yielded by CCDB method resulted in good quality, amplifiability and sufficient quantity for PCR amplification in relatively short extraction time and medium price, showing the CCDB method could be the most suitable method for extracting DNA from wood samples.

Because of high degradation of DNA and abundance of phenolic and lignin compounds in wood logs and commercial wood products, an ideal DNA barcode for species discrimination of wood samples should be short and easy to be amplified, with sufficient information for species discrimination^38^. Previous studies have evaluated different potential DNA barcodes for species discrimination of each of the three genera and found multi-loci combination ITS2 + *trnL*-*trnF* for *Aquilaria*^20^, ITS2 + *trnH*-*psbA* for *Dalbergia*^18^, and ITS2 + *matK* + *ndhF*-*rpl32*^19^ for *Pterocarpus* showed highest success rate in species discrimination of these genera. In this study, to increase the amplification success rate with degraded wood DNA, DNA mini-barcoding approach was adopted and primer sets with amplicon sizes of around 180–300 bp were selected or designed to target each DNA barcode region. Using mini-DNA barcodes, our results are consistent with those of previous studies. In general, the species of each genus were clustered into separate clades with multi-loci combination which has highest species discrimination success rate. Nuclear ribosomal DNA region ITS2 resulted in high PCR amplification success rate and the best species discrimination performance among all the barcodes for all three genera. Since ITS2 generally resulted in the highest species discrimination success rate in single DNA barcode region analysis, it is not surprising that multi-loci combinations included ITS2 barcode regions generally resulted in higher species discrimination success rate comparing with other combinations excluding ITS2. For chloroplast DNA region *matK*, as a core DNA barcode for plant, it generally has a lower PCR amplification success rate and less efficient in species discrimination performance. However, when *matK* combined with ITS2, it helped to yield a better species discrimination performance*.* For example, for *Pterocarpus*, the combination of ITS2 + *matK* successfully resolved all three *Pterocarpus* wood samples; for *Aquilaria*, although ITS2 + *trnL*-*trnF* were sufficient for species discrimination in our study and previous studies^21^, the addition of *matK* helped discrimination between *Gyrinops* sp. and *Aquilaria* sp. by separating them into different clades. Similar situation was also found with *ndhF*-*rpl32* for *Pterocarpus*. Although ITS2 + *matK* were sufficient for species discrimination, addition of *ndhF*-*rpl32* had increased the bootstrap value of the clades of different *Pterocarpus* species and helped better discrimination between *P. macrocarpus* and *P. tinctorius.* The use of multi-loci combination successfully increased the species discrimination success rate of wood samples by providing better species discrimination performance in phylogenetic tree analysis.

For amplification of commercial wood products, although the internal control was successfully amplified in all 10 commercial products, the rate of successful amplification of some barcode regions decreased comparing to the results of amplification of authentic wood samples. For example, in *Aquilaria* products, only one sample (T4959—wood chips) was able to give clear amplification with *trnL*-*trnF* region. Although previous studies have shown that *trnL*-*trnF* region is suitable for identifying agarwood products, highly processed powder and incense stick may have high degree of DNA loss during processing, leading to failure in amplification. This shows the form of products and the degree of processing also affect the DNA content and the amplification. In contrast, designed primers of mini-DNA barcodes enable the PCR amplification in relatively shorter amplicon size and the subsequent utilization of nucleotide sequences from processed wood products. As in the case of *Aquilaria* wood products, *matK* was amplified from all samples using the designed primers AM F11 and AM R5 with amplicon size of around 209 bp (Fig. 2a and Table S2). Also, *matK* was successfully amplified from all *Pterocarpus* wood products using the designed primer set PM F6 & PM-R with amplicon size of around 200 bp (Fig. 2c and Table S2). Based on the result of PCR amplification and phylogenetic analyses, multi-loci combinations were suggested to be amplified for discriminating *Aquilaria*, *Dalbergia* and *Pterocarpus* species from commercial wood products using the species-specific primers designed in this study (Table S2). Having the highest PCR amplification rate and the best species discrimination performance, ITS2 is the main locus for species discrimination. Several loci can act as auxiliaries in increasing the discriminating power, including *matK* and *trnL*-*trnF* for *Aquilaria* species, and *matK* and *trnL* for *Dalbergia* and *Pterocarpus* species. However, further investigation is needed for improving PCR amplification and species discrimination using multi-loci combination for effective authentication of processed wood products.

Although most samples were able to be identified to a single species, for sample T5300 (incense stick formed by powder) and T5339 (powder), they were found to be the closest to *A. crassna* and *A. subintegra* with multi-loci combination ITS2 + *matK*. Previous research have shown that these two species share a very high nucleotide similarity in *matK*, ITS2, *trnL*-*trnF*, *rbcL* and *trnH*-*psbA*^20,39^ and they are hardly discriminable based on their nucleotide sequences. However, these two samples are believed to be sourced from *A. crassana* instead of *A. subintegra* as *A. crassna* is the most commonly exported in form of powder followed by chips according to CITES Trade Database analysis between 1996 and 2015 while the distribution of *A. subintegra* is limited to Thailand and planted in scattered areas in Malaysia with limited trade data^40,41^.

Looking into the accuracy of labelling of products, the mislabelled products all belonged to rosewood (*Dalbergia* or *Pterocarpus*). All three *Aquilaria* products were correctly labelled. This might be because the *Aquilaria* products were labelled up to genus level only. For sample T5204 (labelled as *D. nigra*) and T5205 (labelled as *Dalbergia* spp*.*), they were found to be *D. melanoxylon* and *P. indicus* respectively. *D. nigra, D. melanoxylon*, and *P. indicus* are CITES Appendix I-listed species*,* CITES Appendix II-listed species, and non-CITES protected species, respectively. Since rosewood, especially *Dalbergia* wood, has a high commercial value, mislabelling or adulteration was not surprising. Mislabelling of rosewood product fails to provide accurate information to the consumers and could lead to unsuitable level of law enforcement.

In this work, we have provided effective method for species identification and product authentication of rosewood and agarwood using appropriate DNA extraction method and DNA barcoding techniques. DNA extraction protocols established were able to extract DNA from both heartwood and sapwood of timber logs, as well as processed commercial wood products. For commercial wood products, the rate of mislabelling was moderate (66.67%). All agarwood products were labelled correctly while mislabelling of rosewood in different species or genus were found. Our work can contribute to international timber trade control and extended to other CITES-listed tree species and processed products. We also expect that our DNA extraction protocol will serve as a model for DNA extraction for other heartwood timber and processed wood products.

## Supplementary Information

Supplementary Information.

## Data Availability

All mini-DNA barcode sequences obtained from leaf or twig samples of *Aquilaria*, *Dalbergia* and *Pterocarpus* species with voucher specimens were submitted to and available in the GenBank Database (https://www.ncbi.nlm.nih.gov/nuccore). Accession numbers: OM475713–OM475717, OM478471–OM478475, OM501096–OM501120, OM618755–OM618775, OM648046–OM648087, OM937857–OM937877 (Table S1).
