# Supplementary material for: Comparison of DNA extraction methods on CITES-listed timber species and application in species authentication of commercial products using DNA barcoding
Source: Sci Rep. 2023 Jan 4;13:151. doi: 10.1038/s41598-022-27195-7 (PMC9813357; doi:10.1038/s41598-022-27195-7)
Supplement: Supplementary file 1 — Supplementary Information. [file 41598_2022_27195_MOESM1_ESM.docx]

**Table S1** **| Details of reference leaf and twig samples collected**

| **Genus** | **Species** | **Sample code** | **Collection number/**  **voucher number** | **Type of sample** | **Region of origin** | **GenBank accession number** | | | | |
| --- | --- | --- | --- | --- | --- | --- | --- | --- | --- | --- |
|  |  |  |  |  |  | **ITS2** | ***matK*** | ***trnL-trnF*** |  | |
| *Aquilaria* | *Aquilaria sinensis* | T5160 | T. W. Lau 162 (CUSLSH0596) | Leaf | Hong Kong, China | OM475713 | OM501096 | OM501101 |  | |
|  |  | T5161 | T. W. Lau 171 (CUSLSH0609) | Leaf | Hong Kong, China | OM475714 | OM501097 | OM501102 |  | |
|  |  | T5162 | T. W. Lau 186 (CUSLSH0769) | Leaf | Hong Kong, China | OM475715 | OM501098 | OM501103 |  | |
|  |  | T5198 | KFBG_9203 | Leaf | Hong Kong, China | OM475716 | OM501099 | OM501104 |  | |
|  |  | T5191 | - | Leaf | Hong Kong, China | OM475717 | OM501100 | OM501105 |  | |
|  |  |  |  |  |  | **ITS2** | ***matK*** | ***trnH-psbA*** | | ***trnL*** |
| *Dalbergia* | *Dalbergia assamica* | T5169 | T. Y. Siu 190 (CUSLSH2088) | Leaf | Hong Kong, China | OM618755 | OM648046 | OM937857 | | OM648067 |
|  |  | T5199 | KFBG_7521 | Leaf | Hong Kong, China | OM618756 | OM648047 | OM937858 | | OM648068 |
|  |  | T5211 | T. W. Lau 242 (CUSLSH2171) | Leaf | Hong Kong, China | OM618757 | OM648048 | OM937859 | | OM648069 |
|  |  | T5298 5217 | T. Y. Siu 290 (CUSLSH2252) | Leaf | Hong Kong, China | OM618758 | OM648049 | OM937860 | | OM648070 |
|  | *Dalbergia benthamii* | T5163 | S. K. Tsang 070 (CUSLSH1909) | Leaf | Hong Kong, China | OM618759 | OM648050 | OM937861 | | OM648071 |
|  |  | T5209 | T. Y. Siu 233 (CUSLSH2154) | Leaf | Hong Kong, China | OM618760 | OM648051 | OM937862 | | OM648072 |
|  |  | T5295 5214 | T. Y. Siu 256 (CUSLSH2204) | Leaf | Hong Kong, China | OM618761 | OM648052 | OM937863 | | OM648073 |
|  |  | T5299 5218 | Hu_But_20533 | Leaf | Hong Kong, China | OM618762 | OM648053 | OM937864 | | OM648074 |
|  | *Dalbergia candenatensis* | T5168 | Y. M. Chan 040 (CUSLSH2087) | Leaf | Hong Kong, China | OM618763 | OM648054 | OM937865 | | OM648075 |
|  |  | T5296 5215 | T. Y. Siu 279 (CUSLSH2241) | Leaf | Hong Kong, China | OM618764 | OM648055 | OM937866 | | OM648076 |
|  |  | T5297 5216 | T. Y. Siu 287 (CUSLSH2249) | Leaf | Hong Kong, China | OM618765 | OM648056 | OM937867 | | OM648077 |
|  | *Dalbergia hancei* | T5164 | T. Y. Siu 161 (CUSLSH1947) | Leaf | Hong Kong, China | OM618766 | OM648057 | OM937868 | | OM648078 |
|  |  | T5167 | T. Y. Siu 176 (CUSLSH2035) | Leaf | Hong Kong, China | OM618767 | OM648058 | OM937869 | | OM648079 |
|  |  | T5172 | T. W. Lau 240 (CUSLSH2130) | Leaf | Hong Kong, China | OM618768 | OM648059 | OM937870 | | OM648080 |
|  | *Dalbergia millettii* | T5055 | - | Twig | Hong Kong, China | OM618769 | OM648060 | OM937871 | | OM648081 |
|  |  | T5165 | T. Y. Siu 174 (CUSLSH2033) | Leaf | Hong Kong, China | OM618770 | OM648061 | OM937872 | | OM648082 |
|  |  | T5166 | T. Y. Siu 175 (CUSLSH2034) | Leaf | Hong Kong, China | OM618771 | OM648062 | OM937873 | | OM648083 |
|  |  | T5210 | T. Y. Siu 234 (CUSLSH2155) | Leaf | Hong Kong, China | OM618772 | OM648063 | OM937874 | | OM648084 |
|  | *Dalbergia odorifera* | T5171 | T. Y. Siu 201 (CUSLSH2113) | Leaf | Hong Kong, China | OM618773 | OM648064 | OM937875 | | OM648085 |
|  |  | T5207 | T. Y. Siu 224 (CUSLSH2145) | Leaf | Hong Kong, China | OM618774 | OM648065 | OM937876 | | OM648086 |
|  |  | T5294 5213 | T. Y. Siu 251 (CUSLSH2196) | Leaf | Hong Kong, China | OM618775 | OM648066 | OM937877 | | OM648087 |
|  |  |  |  |  |  | **ITS2** | ***matK*** | ***rbcL*** | | ***ndhF-rpl32*** |
| *Pterocarpus* | *Pterocarpus indicus* | T5208 | T. Y. Siu 225 (CUSLSH2146) | Leaf | Hong Kong, China | OM478471 | OM501106 | OM501116 | | OM501111 |
|  |  | T5290 | T. Y. Siu 565 (CUSLSH2656) | Leaf | Hong Kong, China | OM478472 | OM501107 | OM501117 | | OM501112 |
|  |  | T5291 | T. Y. Siu 564 (CUSLSH2655) | Leaf | Hong Kong, China | OM478473 | OM501108 | OM501118 | | OM501113 |
|  |  | T5292 | T. Y. Siu 563 (CUSLSH2654) | Leaf | Hong Kong, China | OM478474 | OM501109 | OM501119 | | OM501114 |
|  |  | T5293 | T. W. Lau 243 (CUSLSH2172) | Leaf | Hong Kong, China | OM478475 | OM501110 | OM501120 | | OM501115 |

**Table S2** **| Primers and reaction conditions for PCR amplification**

| **Target** | **DNA region** | **Primer set** | **Primer name** | **Sequence (5’ to 3’)** | **Amplicon size** | **Reaction temperature and time** | **Sample Applied** | **Reference** |
| --- | --- | --- | --- | --- | --- | --- | --- | --- |
| *Aquilaria* | ITS2 | ITS-S2F+S3R | ITS-S2F | ATGCGATACTTGGTGTGAAT | 500 bp | 94°C 5 min; 40 cycles of 94°C 30 s, 45°C 45s, 72°C 45 s; 72°C 10 min | Leaf and twig | Lee et al., 2016 |
|  |  |  | ITS-S3R | GACGCTTCTCCAGACTACAAT |  |  |  |  |
|  |  | AI F6R10 | AI F6 | ATGTCTGTGAGGGCTGTGTG | 181 bp | 94°C 5 min; 40 cycles of 94°C 30 s, 45°C 45s, 72°C 45 s; 72°C 10 min | Wood and commercial product | This study |
|  |  |  | AI R10 | CCGGGGTTGTCCACGAAACA |  |  |  |  |
|  | *trnL-trnF* | AT | trnL-F | GGTTCAAGTCCCTCTATCCC | 471 bp | 94°C 5 min; 40 cycles of 94°C 30 s, 45°C 45s, 72°C 45 s; 72°C 10 min | Leaf and twig | Jiao et al., 2014 |
|  |  |  | trnL-R | TCTGCTCTACCAGCTGTGCT |  |  |  |  |
|  |  | AT F2R1 | AT F2 | TTTTGTTAGTGGTTCAAAG | 280 bp | 94°C 5 min; 40 cycles of 94°C 30 s, 45°C 45s, 72°C 45 s; 72°C 10 min | Wood and commercial product | This study |
|  |  |  | AT R1 | TCAAAATAAAATGAAAAAAAAA |  |  |  |  |
|  | *matK* | AM | matK-F | AAGCCTTTCGCTGCTGGTT | 470 bp | 94°C 5 min; 40 cycles of 94°C 30 s, 45°C 45s, 72°C 45 s; 72°C 10 min | Leaf and twig | Jiao et al., 2014 |
|  |  |  | matK-R | ACGGACCCCTCCTGATTGA |  |  |  |  |
|  |  | AM F11R5 | AM F11 | GGTTGTTCTGAAAATCATTA | 209 bp | 94°C 5 min; 40 cycles of 94°C 30 s, 45°C 45s, 72°C 45 s; 72°C 10 min | Wood and commercial product | This study |
|  |  |  | AM R5 | AATCCATTTCTATTTTGAAT |  |  |  |  |
| *Dalbergia* | *trnH-psbA* | DP | TP-F | CTTCCCTCTAGACCTAGCTTCG | 334 bp | 94°C 5 min; 40 cycles of 94°C 30 s, 45°C 45s, 72°C 45 s; 72°C 10 min | Leaf, twig, wood, and commercial product | Yu et al., 2017 |
|  |  |  | TP-R | TTGGCTACATCCGCCCTT |  |  |  |  |
|  | *trnL* | DT | trnL-F | CTGAAAGTAAAGAAAAGT | 314 bp | 94°C 5 min; 40 cycles of 94°C 30 s, 40°C 45s, 72°C 45 s; 72°C 10 min | Leaf, twig, wood, and commercial product | Yu et al., 2017 |
|  |  |  | trnL-R | TATGATGGAGTGAATGAT |  |  |  |  |
|  | *matK* | DM | matK-F | GTCTTCGATACTGGGTGAA | 467 bp | 94°C 5 min; 40 cycles of 94°C 30 s, 45°C 45s, 72°C 45 s; 72°C 10 min | Leaf and twig | Yu et al., 2017 |
|  |  |  | matK-R | GATCGTTCCAGGTTGAGA |  |  |  |  |
|  |  | DM F2R2 | DM F2 | GTGAACACGAATCCATCTTCCTT | 300 bp | 94°C 5 min; 40 cycles of 94°C 30 s, 40°C 45s, 72°C 45 s; 72°C 10 min | Wood and commercial product | This study |
|  |  |  | DM R2 | CGTTCCGGGTTGAGACCAAA |  |  |  |  |

*Continued*

**Table S2** **| Continued**

| **Target** | **DNA region** | **Primer set** | **Primer name** | **Sequence (5’ to 3’)** | **Amplicon size** | **Reaction temperature and time** | **Sample Applied** | **Reference** |
| --- | --- | --- | --- | --- | --- | --- | --- | --- |
| *Dalbergia*  and  *Pterocarpus* | ITS2 | DAPT ITS2 | ITS2-F | ATGCGATACTTGGTGTGA | 348 bp | 94°C 5 min; 40 cycles of 94°C 30 s, 45°C 45s, 72°C 45 s; 72°C 10 min | Leaf and twig | Yu et al., 2017  Jiao et al., 2018 |
|  |  |  | ITS2-R | TAGCCCCGCCTGAACCTGA |  |  |  |  |
|  |  | DAPT ITS2 F3R3 | DAPT ITS2 F3 | CCCGTGAACCATCGAGTCTT | 180 bp | 94°C 5 min; 40 cycles of 94°C 30 s, 40°C 45s, 72°C 45 s; 72°C 10 min | Wood and commercial product | This study |
|  |  |  | DAPT ITS2 R3 | TCCACCACGAACCCGATTTT |  |  |  |  |
| *Pterocarpus* | *ndhF-rpl32* | PN | NR-F | GATTCGTAATGAGTTTTGTTTC | 167-173 bp | 94°C 5 min; 40 cycles of 94°C 30 s, 40°C 45s, 72°C 45 s; 72°C 10 min | Leaf, twig, wood, and commercial product | Jiao et al., 2018 |
|  |  |  | NR-R | AGGAATTTGTTTATTGGACC |  |  |  |  |
|  | *matK* | PM | matK-F | TTTCGTCTACCTTATCCTTCTTCA | 239 bp | 94°C 5 min; 40 cycles of 94°C 30 s, 45°C 45s, 72°C 45 s; 72°C 10 min | Leaf and twig | Jiao et al., 2018 |
|  |  |  | matK-R | CTAGCATTTGACTCCGTACCAC |  |  |  |  |
|  |  | PM F6 & PM-R | PM F6 | AAGGAAAATCCATTCTGGCT | 200 bp | 94°C 5 min; 40 cycles of 94°C 30 s, 40°C 45s, 72°C 45 s; 72°C 10 min | Wood and commercial product | This study |
|  |  |  | matK-R | CTAGCATTTGACTCCGTACCAC |  |  |  |  |
|  | *rbcL* | PR | rbcL-F  rbcL-R | TGCCGAATCTTCTACTGG | 485 bp | 94°C 5 min; 40 cycles of 94°C 30 s, 45°C 45s, 72°C 45 s; 72°C 10 min | Leaf and twig | Jiao et al., 2018 |
|  |  |  |  | ATAAGAAACGGTCCCTCC |  |  |  |  |
|  |  | PR F7R3 | PR F7 | CCGATGGGCTTACCAGTCTT | 259 bp | 94°C 5 min; 40 cycles of 94°C 30 s, 40°C 45s, 72°C 45 s; 72°C 10 min | Wood and commercial product | This study |
|  |  |  | PR R3 | AACTTGGATACCGTGAGGCG |  |  |  |  |
| Internal control | p6 loop of *trnL(UAA)* | trnL gh | trnL-g | GGGCAATCCTGAGCCAA | 10-143 bp | 94°C 5 min; 40 cycles of 94°C 30 s, 40°C 45s, 72°C 45 s; 72°C 10 min | Leaf, twig, wood, and commercial product | Taberlet et al., 2007 |
|  |  |  | trnL-h | CCATTGAGTCTCTGCACCTATC |  |  |  |  |

**Table S3** **| Barcode sequences downloaded from NCBI GenBank for library development for the identification of *Aquilaria* authentic wood samples and *Aquilaria* commercial products**

| **DNA barcode locus** | **Species** | **GenBank accession** | | | | | |
| --- | --- | --- | --- | --- | --- | --- | --- |
| ITS2 | *Aquilaria agallocha* | MH134137 |  |  |  |  |  |
|  | *Aquilaria beccariana* | MH134138 |  |  |  |  |  |
|  | *Aquilaria crassna* | KU244108 | KU244109 | KU244110 | KU244111 | KY817942 | KY817943 |
|  |  | KY817944 | KY817968 | KY817969 |  |  |  |
|  | *Aquilaria hirta* | KU244112 | KU244113 | KU244114 | MH134141 |  |  |
|  | *Aquilaria malaccensis* | KU244115 | KU244116 | KU244117 | MH134142 |  |  |
|  | *Aquilaria microcarpa* | KU244118 | KU244119 | KU244120 | MH134143 |  |  |
|  | *Aquilaria rostrata* | MH134144 |  |  |  |  |  |
|  | *Aquilaria rugosa* | MH134145 |  |  |  |  |  |
|  | *Aquilaria sinensis* | KU244121 | KU244122 | KU244123 | KU244124 | KU244125 | KU244126 |
|  |  | KY817974 | KY817975 | KY817976 | MH134146 |  |  |
|  | *Aquilaria subintegra* | KU244127 | KU244128 | MH134147 |  |  |  |
|  | *Aquilaria yunnanensis* | KU244129 | KU244130 | KU244131 | KY817983 | KY817984 | KY817985 |
|  |  | MH134148 |  |  |  |  |  |
|  | *Gyrinops caudata* | MH134149 |  |  |  |  |  |
|  | *Gyrinops versteegii* | MH134150 |  |  |  |  |  |
|  | *Gyrinops walla* | MH134151 |  |  |  |  |  |
|  | *Gonystylus bancanus* | MH134152 |  |  |  |  |  |
|  | *Phaleria macrocarpa* | MH134153 |  |  |  |  |  |
|  | *Wikstroemia ridleyi* | MH134154 |  |  |  |  |  |
| *matK* | *Aquilaria agallocha* | MF443398 |  |  |  |  |  |
|  | *Aquilaria beccariana* | MF443399 |  |  |  |  |  |
|  | *Aquilaria crassna* | KU244186 | KU244187 | KU244188 | KU244189 | KY927297 | KY927298 |
|  |  | KY927299 | KY927323 | KY927324 |  |  |  |
|  | *Aquilaria hirta* | KU244190 | KU244191 | KU244192 |  |  |  |
|  | *Aquilaria malaccensis* | KU244193 | KU244194 | KU244195 |  |  |  |
|  | *Aquilaria microcarpa* | KU244196 | KU244197 | KU244198 |  |  |  |
|  | *Aquilaria rostrata* | MF443401 |  |  |  |  |  |
|  | *Aquilaria rugosa* | MF443402 |  |  |  |  |  |
|  | *Aquilaria sinensis* | KU244199 | KU244200 | KU244201 | KU244202 | KU244203 | KU244204 |
|  |  | KY927329 | KY927330 | KY927331 |  |  |  |
|  | *Aquilaria subintegra* | KU244205 | KU244206 |  |  |  |  |
|  | *Aquilaria yunnanensis* | KU244207 | KU244208 | KU244209 | KY927338 | KY927339 | KY927340 |
|  | *Gyrinops caudata* | KU726323 |  |  |  |  |  |
|  | *Gyrinops versteegii* | KU244054 |  |  |  |  |  |
|  | *Gyrinops walla* | MF433431 |  |  |  |  |  |
|  | *Gonystylus bancanus* | KU244055 |  |  |  |  |  |
|  | *Phaleria macrocarpa* | MH134161 |  |  |  |  |  |
|  | *Wikstroemia ridleyi* | MH134162 |  |  |  |  |  |
| *trnL–trnF* | *Aquilaria agallocha* | MF443428 |  |  |  |  |  |
|  | *Aquilaria beccariana* | MF443429 |  |  |  |  |  |
|  | *Aquilaria crassna* | KU244030 | KU244031 | KU244032 | KU244033 | KY927209 | KY927210 |
|  |  | KY927211 | KY927235 | KY927236 |  |  |  |
|  | *Aquilaria hirta* | KU244034 | KU244035 | KU244036 |  |  |  |
|  | *Aquilaria malaccensis* | KU244037 | KU244038 | KU244039 |  |  |  |
|  | *Aquilaria microcarpa* | KU244040 | KU244041 | KU244042 |  |  |  |
|  | *Aquilaria rostrata* | KT364475 |  |  |  |  |  |
|  | *Aquilaria rugosa* | MF443440 |  |  |  |  |  |
|  | *Aquilaria sinensis* | KU244043 | KU244044 | KU244045 | KU244046 | KU244047 | KU244048 |
|  |  | KY927241 | KY927242 | KY927243 |  |  |  |
|  | *Aquilaria subintegra* | KU244049 | KU244050 | KU244049 |  |  |  |
|  | *Aquilaria yunnanensis* | KU244051 | KU244052 | KU244053 | KY927250 | KY927251 | KY927252 |
|  | *Gyrinops caudata* | MF443403 |  |  |  |  |  |
|  | *Gyrinops versteegii* | KU244210 |  |  |  |  |  |
|  | *Gyrinops walla* | MF443404 |  |  |  |  |  |
|  | *Gonystylus bancanus* | KU244211 |  |  |  |  |  |
|  | *Phaleria macrocarpa* | MH134155 |  |  |  |  |  |
|  | *Wikstroemia ridleyi* | MH134156 |  |  |  |  |  |

**Table S4** **| Barcode sequences downloaded from NCBI GenBank for library development for the identification of *Dalbergia* authentic wood samples and *Dalbergia* commercial products**

| **DNA barcode locus** | **Species** | **GenBank accession** | | | | | |
| --- | --- | --- | --- | --- | --- | --- | --- |
| ITS2 | *Dalbergia assamica* | KM521354 | KM521356 |  |  |  |  |
|  | *Dalbergia cana* | KM521358 | KM521357 |  |  |  |  |
|  | *Dalbergia candenatensis* | KM521359 | KM521360 | KM521362 | KM521361 |  |  |
|  | *Dalbergia cochinchinensis* | KM521363 | KM521364 | KM521365 | KM521366 | KM521367 | KY484185 |
|  |  | KY484186 | KY484187 | KY484188 | KY484189 |  |  |
|  | *Dalbergia cultrata* | KM521369 | KM521371 | KM521370 |  |  |  |
|  | *Dalbergia dyeriana* | KM521372 |  |  |  |  |  |
|  | *Dalbergia ecastaphyllum* | KM521373 | KM521374 |  |  |  |  |
|  | *Dalbergia entadoides* | KM521375 |  |  |  |  |  |
|  | *Dalbergia hainanensis* | KY484181 | KY484182 | KY484183 | KY484184 |  |  |
|  | *Dalbergia hupeana* | KM521378 | KY484173 | KY484174 | KY484175 | KY484176 | KY484177 |
|  |  | KY484178 | KY484179 | KY484180 |  |  |  |
|  | *Dalbergia lanceolaria* | KM521379 | KM521380 |  |  |  |  |
|  | *Dalbergia latifolia* | KY484190 | KY484191 | KY484192 | KY484193 | KM521381 |  |
|  | *Dalbergia melanoxylon* | KM521382 | KY484194 | KY484195 | KY484196 | KY484197 |  |
|  | *Dalbergia miscolobium* | KM521383 |  |  |  |  |  |
|  | *Dalbergia monetaria* | KM521384 | KM521385 |  |  |  |  |
|  | *Dalbergia nigrescens* | KM521386 | KM521387 |  |  |  |  |
|  | *Dalbergia odorifera* | KM521389 | KM521388 | KM521377 | KY484164 | KY484165 | KY484166 |
|  |  | KY484167 | KY484168 | KY484169 | KY484170 | KY484171 | KY484172 |
|  | *Dalbergia oliveri* | KM521390 | KM521391 | KM521392 | KM521393 | KM521394 | KM521395 |
|  |  | KM521396 | KM521397 | FR854133 | FR854134 | FR854135 | FR854136 |
|  |  | FR854137 | KY484198 | KY484199 | KY484200 |  |  |
|  | *Dalbergia ovata* | KM521398 | KM521399 |  |  |  |  |
|  | *Dalbergia pinnata* | KM521400 | KM521401 |  |  |  |  |
|  | *Dalbergia retusa* | KY484201 | KY484202 | KY484203 | KY484204 | KY484205 |  |
|  | *Dalbergia rimosa* | KM521402 | KM521403 | KM521404 | KM521405 | KM521376 |  |
|  | *Dalbergia sericea* | KM521406 |  |  |  |  |  |
|  | *Dalbergia sissoo* | KM521407 | KM521408 | KM521409 | KX057860 |  |  |
|  | *Dalbergia stevensonii* | KY484206 | KY484207 | KY484208 |  |  |  |
|  | *Dalbergia stipulacea* | KM521410 | KM521411 | KM521412 | KM521413 |  |  |
|  | *Dalbergia subcymosa* | KM521414 |  |  |  |  |  |
|  | *Dalbergia trichocarpa* | KM521415 | KM521416 |  |  |  |  |
|  | *Dalbergia velutina* | KM521417 | KM521418 | KM521419 | KM521420 |  |  |
|  | *Machaerium lunatum* | KM521421 |  |  |  |  |  |
|  | *Machaerium salvadorense* | KM521422 |  |  |  |  |  |

Continued

**Table S4** **| Continued**

| **DNA barcode locus** | **Species** | **GenBank accession** | | | | | |
| --- | --- | --- | --- | --- | --- | --- | --- |
| *matK* | *Dalbergia assamica* | KM521275 | KM521273 | KM521272 |  |  |  |
|  | *Dalbergia benthamii* | KM521278 |  |  |  |  |  |
|  | *Dalbergia cana* | KM521284 | KM521279 |  |  |  |  |
|  | *Dalbergia cochinchinensis* | KM521288 | KM521286 | KM521289 | KM521326 | KM521285 | KM521287 |
|  |  | KY484226 | KY484227 | KY484228 | KY484229 |  |  |
|  | *Dalbergia cultrata* | KM521300 | KM521292 | KM521290 | KM521291 |  |  |
|  | *Dalbergia dyeriana* | KM521295 |  |  |  |  |  |
|  | *Dalbergia ecastaphyllum* | KM521296 | KM521297 |  |  |  |  |
|  | *Dalbergia entadoides* | KM521298 |  |  |  |  |  |
|  | *Dalbergia hainanensis*  *Dalbergia hancei* | KY484223 | KY484224 | KY484225 |  |  |  |
|  |  | KM521303 | KM521302 |  |  |  |  |
|  | *Dalbergia hupeana* | KM521305 | KM521306 | KY484216 | KY484217 | KY484218 | KY484219 |
|  |  | KY484220 | KY484221 | KY484222 |  |  |  |
|  | *Dalbergia lanceolaria* | KM521307 | KM521270 | KM521308 |  |  |  |
|  | *Dalbergia latifolia* | KY484230 | KY484231 | KY484232 | KM521309 |  |  |
|  | *Dalbergia melanoxylon* | KM521310 | KY484233 | KY484234 | KY484235 |  |  |
|  | *Dalbergia mimosoides* | KM521311 |  |  |  |  |  |
|  | *Dalbergia miscolobium*  *Dalbergia monetaria* | KM521312 |  |  |  |  |  |
|  |  | KM521313 | KM521314 |  |  |  |  |
|  | *Dalbergia nigrescens* | KM521317 | KM521318 | KM521315 | KM521316 |  |  |
|  | *Dalbergia odorifera* | KM521320 | KM521319 | KM521301 | KY484209 | KY484210 | KY484211 |
|  |  | KY484212 | KY484213 | KY484214 | KY484215 |  |  |
|  | *Dalbergia oliveri* | KM521277 | KM521276 | KM521325 | KM521321 | KM521323 | KM521294 |
|  |  | KM521324 | KM521322 | FR854108 | FR854109 | FR854110 | FR854111 |
|  |  | FR854112 | KY484236 | KY484237 |  |  |  |
|  | *Dalbergia ovata* | KM521327 | KM521328 |  |  |  |  |
|  | *Dalbergia pinnata* | KM521329 | KM521330 |  |  |  |  |
|  | *Dalbergia retusa* | KY484238 | KY484239 | KY484240 | KY484241 |  |  |
|  | *Dalbergia rimosa* | KM521271 | KM521333 | KM521332 | KM521334 | KM521299 |  |
|  | *Dalbergia sericea* | KM521336 |  |  |  |  |  |
|  | *Dalbergia sissoo* | KM521337 | KM521338 |  |  |  |  |
|  | *Dalbergia stevensonii* | KY484242 |  |  |  |  |  |
|  | *Dalbergia stipulacea* | KM521339 | KM521340 | KM521341 |  |  |  |
|  | *Dalbergia subcymosa* | KM521342 |  |  |  |  |  |
|  | *Dalbergia trichocarpa* | KM521343 | KM521344 |  |  |  |  |
|  | *Dalbergia velutina* | KM521335 | KM521345 | KM521348 | KM521346 | KM521347 |  |
|  | *Dalbergia volubilis* | KM521350 | KM521349 |  |  |  |  |
|  | *Machaerium lunatum* | KM521425 |  |  |  |  |  |
|  | *Machaerium salvadorense* | KM521352 |  |  |  |  |  |

Continued

**Table S4** **| Continued**

| **DNA barcode locus** | **Species** | **GenBank accession** | | | | | |
| --- | --- | --- | --- | --- | --- | --- | --- |
| *trnL* | *Dalbergia cochinchinensis* | KY484262 | KY608877 | KY484263 | KY484264 | KY484265 |  |
|  | *Dalbergia hainanensis* | KY484258 | KY484259 | KY484260 | KY484261 |  |  |
|  | *Dalbergia hupeana* | KY484251 | KY484252 | KY484253 | KY484254 | KY484255 | KY484256 |
|  |  | KY484257 |  |  |  |  |  |
|  | *Dalbergia latifolia* | KY484266 | KY484267 |  |  |  |  |
|  | *Dalbergia melanoxylon* | KY484268 | KY484269 | KY484270 | KY484271 |  |  |
|  | *Dalbergia odorifera* | KY484243 | KY484244 | KY484245 | KY484246 | KY484247 | KY484248 |
|  |  | KY484249 | KY484250 |  |  |  |  |
|  | *Dalbergia oliveri* | FR854183 | FR854184 | FR854185 | FR854186 | FR854187 | KY484272 |
|  |  | KY484273 | KY484274 | KY484275 |  |  |  |
|  | *Dalbergia retusa* | KY484276 | KY484277 | KU663046 | KY484278 | KY484279 |  |
|  | *Dalbergia sissoo* | KX268174 |  |  |  |  |  |
|  | *Dalbergia stevensonii* | KY484280 | KY484281 |  |  |  |  |
| *trnH-psbA* | *Dalbergia cochinchinensis* | KY484389 | KY484390 | KY484391 | KY484392 | KY484393 |  |
|  | *Dalbergia hainanensis* | KY484385 | KY484386 | KY484387 | KY484388 |  |  |
|  | *Dalbergia hupeana* | KY484377 | KY484378 | KY484379 | KY484380 | KY484381 | KY484382 |
|  |  | KY484383 | KY484384 |  |  |  |  |
|  | *Dalbergia latifolia* | KY484394 | KY484395 | KY484396 | KY484397 | KY484398 |  |
|  | *Dalbergia melanoxylon* | KY484399 | KY484400 | KY484401 | KY484402 |  |  |
|  | *Dalbergia odorifera* | KY484368 | KY484369 | KY484370 | KY484371 | KY484372 | KY484373 |
|  |  | KY484374 | KY484375 | KY484376 |  |  |  |
|  | *Dalbergia oliveri* | FR854153 | FR854154 | FR854155 | FR854156 | FR854157 | KY484403 |
|  |  | KY484404 | KY484405 | KY484406 |  |  |  |
|  | *Dalbergia retusa* | KY484407 | KY484408 | KY484409 | KY484410 | KY484411 | KY484412 |
|  | *Dalbergia stevensonii* | KY484413 | KY484414 | KY484415 |  |  |  |

**Table S5** **| Barcode sequences downloaded from NCBI GenBank for library development for the identification of *Pterocarpus* authentic wood samples and *Pterocarpus* commercial products**

| **DNA barcode locus** | **Species** | **GenBank accession** | | | | | |
| --- | --- | --- | --- | --- | --- | --- | --- |
| ITS2 | *Pterocarpus angolensis* | KY829137 | KY829138 | KY829139 |  |  |  |
|  | *Pterocarpus indicus* | KY829140 | KY829141 | KY829142 | KY829143 |  |  |
|  | *Pterocarpus macrocarpus* | KY829144 | KY829145 |  |  |  |  |
|  | *Pterocarpus santalinus* | KY829146 | KY829147 | KY829148 | KY829149 | KY829150 | KY829151 |
|  |  | KY829152 | KY829153 |  |  |  |  |
|  | *Pterocarpus soyauxii* | KY829154 | KY829155 |  |  |  |  |
|  | *Pterocarpus tinctorius* | KY829156 | KY829157 | KY829158 | KY829159 | KY829160 | KY829161 |
|  |  | KY829162 |  |  |  |  |  |
| *matK* | *Pterocarpus angolensis* | KY829163 | KY829164 | KY829165 | KY829166 | KY829167 | KY829168 |
|  | *Pterocarpus indicus* | KY829169 | KY829170 | KY829171 | KY829172 | KY829173 |  |
|  | *Pterocarpus macrocarpus* | KY829174 | KY829175 | KY829176 |  |  |  |
|  | *Pterocarpus santalinus* | KY829177 | KY829178 | KY829179 | KY829180 | KY829181 | KY829182 |
|  |  | KY829183 | KY829184 | KY829185 | KY829186 |  |  |
|  | *Pterocarpus soyauxii* | KY829187 | KY829188 |  |  |  |  |
|  | *Pterocarpus tinctorius* | KY829189 | KY829190 | KY829191 | KY829192 | KY829193 | KY829194 |
|  |  | KY829195 |  |  |  |  |  |
| *rbcL* | *Pterocarpus angolensis*  *Pterocarpus indicus*  *Pterocarpus macrocarpus* | KY829233 | KY829234 | KY829235 | KY829236 | KY829237 |  |
|  |  | KY829238 | KY829239 | KY829240 | KY829241 |  |  |
|  |  | KY829242 | KY829243 | KY829244 | KY829245 |  |  |
|  | *Pterocarpus santalinus* | KY829246 | KY829247 | KY829248 | KY829249 | KY829250 |  |
|  | *Pterocarpus soyauxii* | KY829251 | KY829252 |  |  |  |  |
|  | *Pterocarpus tinctorius* | KY829253 | KY829254 | KY829255 | KY829256 | KY829257 | KY829258 |
|  |  | KY829259 |  |  |  |  |  |
| *ndhF-rpl32* | *Pterocarpus angolensis* | KY829196 | KY829197 | KY829198 | KY829199 | KY829200 |  |
|  | *Pterocarpus indicus* | KY829201 | KY829202 | KY829203 | KY829204 | KY829205 | KY829206 |
|  | *Pterocarpus macrocarpus* | KY829207 | KY829208 | KY829209 | KY829210 |  |  |
|  | *Pterocarpus santalinus* | KY829211 | KY829212 | KY829213 | KY829214 | KY829215 | KY829216 |
|  |  | KY829217 | KY829218 | KY829219 | KY829220 | KY829221 |  |
|  | *Pterocarpus soyauxii* | KY829222 | KY829223 | KY829224 | KY829225 |  |  |
|  | *Pterocarpus tinctorius* | KY829226 | KY829227 | KY829228 | KY829229 | KY829230 | KY829231 |
|  |  | KY829232 |  |  |  |  |  |

**Figure S1 | Authentic wood samples used for the comparison of three genomic DNA extraction methods.**

(a)-(e) *Aquilaria*: (a) T5196, (b) T4960, (c) T5149, (d) T5154, (e) T5155; (f)-(i) *Dalbergia*: (f) T4961, (g) T4966, (h) T5152, (i) T5153; (j)-(l) *Pterocarpus* (j) T4964, (k) T4965, (l) T5197.

**Figure S2 | Commercial wood products used for the comparison of three genomic DNA extraction methods.**

(a) T4959 Wood chips, (b) T5300 Powder, (c) T5339 incense stick, (d) T5204 Beads from bracelet, (e) T5348 Wooden disc, (f) T4962 Beads from necklace, (g) T4963 Wood block from furniture part, (h) T5150 Wood block, (i) T5338 Wood piece, (j) T5205 Beads from bracelet.

**Figure S3** **| Neighbour-joining tree constructed from single DNA barcode region, ITS2, for species identification of *Aquilaria* wood samples.**

**Figure S4** **| Neighbour-joining tree constructed from single DNA barcode region, *matK*, for species identification of *Aquilaria* wood samples.**

**Figure S5** **| Neighbour-joining tree constructed from single DNA barcode region, *trnL-trnF*, for species identification of *Aquilaria* wood samples.**

**Figure S6** **| Neighbour-joining tree constructed from multi-loci combination ITS2+*matK* for species identification of *Aquilaria* wood samples.**

**Figure S7** **| Neighbour-joining tree constructed from multi-loci combination ITS2+*trnL-trnF* for species identification of *Aquilaria* wood samples.**

**Figure S8** **| Neighbour-joining tree constructed from multi-loci combination *matK*+*trnL*-*trnF* for species identification of *Aquilaria* wood samples.**

**Figure S9 | Neighbour-joining tree constructed from multi-loci combination ITS2+*matK*+*trnL*-*trnF* for species identification of *Aquilaria* wood samples.**

**Figure S10** **| Neighbour-joining tree constructed from single DNA barcode region, ITS2, for species identification of *Dalbergia* wood samples.**

**Figure S11** **| Neighbour-joining tree constructed from single DNA barcode region, *matK*, for species identification of *Dalbergia* wood samples.**

**Figure S12** **| Neighbour-joining tree constructed from single DNA barcode region, *trnL*, for species identification of *Dalbergia* wood samples.**

**Figure S13** **| Neighbour-joining tree constructed from single DNA barcode region, *trnH-psbA*, for species identification of *Dalbergia* wood samples.**

**Figure S14** **| Neighbour-joining tree constructed from multi-loci combination ITS2+*matK* for species identification of *Dalbergia* wood samples.**

**Figure S15** **| Neighbour-joining tree constructed from multi-loci combination ITS2+*trnH-psbA* for species identification of *Dalbergia* wood samples.**

**Figure S16** **| Neighbour-joining tree constructed from multi-loci combination ITS2+*trnL* for species identification of *Dalbergia* wood samples.**

**Figure S17** **| Neighbour-joining tree constructed from multi-loci combination *matK*+*trnH-psbA* for species identification of *Dalbergia* wood samples.**

**Figure S18** **| Neighbour-joining tree constructed from multi-loci combination *matK*+*trnL* for species identification of *Dalbergia* wood samples.**

**Figure S19** **| Neighbour-joining tree constructed from multi-loci combination *trnH-psbA*+*trnL* for species identification of *Dalbergia* wood samples.**

**Figure S20** **| Neighbour-joining tree constructed from multi-loci combination ITS2+*matK*+*trnH-psbA* for species identification of *Dalbergia* wood samples.**

**Figure S21 | Neighbour-joining tree constructed from multi-loci combination ITS2+*matK*+*trnL* for species identification of *Dalbergia* wood samples.**

**Figure S22 | Neighbour-joining tree constructed from multi-loci combination ITS2+*trnH-psbA*+*trnL* for species identification of *Dalbergia* wood samples.**

**Figure S23 | Neighbour-joining tree constructed from multi-loci combination *matK*+*trnH-psbA*+*trnL* for species identification of *Dalbergia* wood samples.**

**Figure S24 | Neighbour-joining tree constructed from multi-loci combination ITS2+*matK*+*trnH*-*psbA*+*trnL* for species identification of *Dalbergia* wood samples.**

**Figure S25** **| Neighbour-joining tree constructed from single DNA barcode region, ITS2, for species identification of *Pterocarpus* wood samples.**

**Figure S26** **| Neighbour-joining tree constructed from single DNA barcode region, *matK*, for species identification of *Pterocarpus* wood samples.**

**Figure S27** **| Neighbour-joining tree constructed from single DNA barcode region, *rbcL*, for species identification of *Pterocarpus* wood samples.**

**Figure S28 | Neighbour-joining tree constructed from single DNA barcode region, *ndhF-rpl32*, for species identification of *Pterocarpus* wood samples.**

**Figure S29 | Neighbour-joining tree constructed from multi-loci combination ITS2+*matK* for species identification of *Pterocarpus* wood samples.**

**Figure S30 | Neighbour-joining tree constructed from multi-loci combination ITS2+*rbcL* for species identification of *Pterocarpus* wood samples.**

**Figure S31 | Neighbour-joining tree constructed from multi-loci combination ITS2+*ndhF-rpl32* for species identification of *Pterocarpus* wood samples.**

**Figure S32 | Neighbour-joining tree constructed from multi-loci combination *matK*+*rbcL* for species identification of *Pterocarpus* wood samples.**

**Figure S33 | Neighbour-joining tree constructed from multi-loci combination *matK*+*ndhF-rpl32* for species identification of *Pterocarpus* wood samples.**

**Figure S34 | Neighbour-joining tree constructed from multi-loci combination *rbcL*+*ndhF-rpl32* for species identification of *Pterocarpus* wood samples.**

**Figure S35 | Neighbour-joining tree constructed from multi-loci combination ITS2+*matK*+*rbcL* for species identification of *Pterocarpus* wood samples.**

**Figure S36 | Neighbour-joining tree constructed from multi-loci combination ITS2+*matK*+*ndhF-rpl32* for species identification of *Pterocarpus* wood samples.**

**Figure S37 | Neighbour-joining tree constructed from multi-loci combination ITS2+*rbcL*+*ndhF-rpl32* for species identification of *Pterocarpus* wood samples.**

**Figure S38 | Neighbour-joining tree constructed from multi-loci combination *matK*+*rbcL*+*ndhF-rpl32*for species identification of *Pterocarpus* wood samples.**

**Figure S39 | Neighbour-joining tree constructed from multi-loci combination ITS2+*matK*+*rbcL*+*ndhF-rpl32* for species identification of *Pterocarpus* wood samples.**

**Figure S40 | Neighbour-joining tree constructed from single DNA barcode region, ITS2, for species identification of *Aquilaria* commercial wood products.**

**Figure S41 | Neighbour-joining tree constructed from single DNA barcode region, *matK*, for species identification of *Aquilaria* commercial wood products.**

**Figure S42 | Neighbour-joining tree constructed from single DNA barcode region, *trnL*-*trnF*, for species identification of *Aquilaria* commercial wood product.**

**Figure S43 | Neighbour-joining tree constructed from multi-loci combination ITS2+*matK* for species identification of *Aquilaria* commercial wood products.**

**Figure S44 | Neighbour-joining tree constructed from multi-loci combination ITS2+*trnL-trnF* for species identification of *Aquilaria* commercial wood product.**

**Figure S45 | Neighbour-joining tree constructed from multi-loci combination *matK*+*trnL-trnF* for species identification of *Aquilaria* commercial wood product.**

**Figure S46 | Neighbour-joining tree constructed from multi-loci combination ITS2+*matK*+*trnL-trnF* for species identification of *Aquilaria* commercial wood product.**

**Figure S47 | Neighbour-joining tree constructed from single DNA barcode region, ITS2, for species identification of *Dalbergia* commercial wood product.**

**Figure S48 | Neighbour-joining tree constructed from single DNA barcode region, *matK*, for species identification of *Dalbergia* commercial wood product.**

**Figure S49 | Neighbour-joining tree constructed from single DNA barcode region, *trnL*, for species identification of *Dalbergia* commercial wood products.**

**Figure S50 | Neighbour-joining tree constructed from single DNA barcode region, *trnH-psbA*, for species identification of *Dalbergia* commercial wood product.**

**Figure S51 | Neighbour-joining tree constructed from multi-loci combination ITS2+*matK* for species identification of *Dalbergia* commercial wood product.**

**Figure S52 | Neighbour-joining tree constructed from multi-loci combination ITS2+*trnH-psbA* for species identification of *Dalbergia* commercial wood product.**

**Figure S53 | Neighbour-joining tree constructed from multi-loci combination ITS2+*trnL* for species identification of *Dalbergia* commercial wood products.**

**Figure S54 | Neighbour-joining tree constructed from multi-loci combination *matK*+*trnH-psbA* for species identification of *Dalbergia* commercial wood product.**

**Figure S55 | Neighbour-joining tree constructed from multi-loci combination *matK*+*trnL* for species identification of *Dalbergia* commercial wood product.**

**Figure S56 | Neighbour-joining tree constructed from multi-loci combination *trnH-psbA*+*trnL* for species identification of *Dalbergia* commercial wood product.**

**Figure S57 | Neighbour-joining tree constructed from multi-loci combination ITS2+*matK*+*trnH-psbA* for species identification of *Dalbergia* commercial wood product.**

**Figure S58 | Neighbour-joining tree constructed from multi-loci combination ITS2+*matK*+*trnL* for species identification of *Dalbergia* commercial wood product.**

**Figure S59 | Neighbour-joining tree constructed from multi-loci combination ITS2+*trnH-psbA*+*trnL* for species identification of *Dalbergia* commercial wood product.**

**Figure S60 | Neighbour-joining tree constructed from multi-loci combination *matK*+*trnH-psbA*+*trnL* for species identification of *Dalbergia* commercial wood product.**

**Figure S61 | Neighbour-joining tree constructed from multi-loci combination ITS2+*matK*+*trnH-psbA*+*trnL* for species identification of *Dalbergia* commercial wood product.**

**Figure S62 | Neighbour-joining tree constructed from single DNA barcode region, ITS2, for species identification of *Pterocarpus* commercial wood products.**

**Figure S63 | Neighbour-joining tree constructed from single DNA barcode region, *matK*, for species identification of *Pterocarpus* commercial wood products.**

**Figure S64 | Neighbour-joining tree constructed from single DNA barcode region, *rbcL*, for species identification of *Pterocarpus* commercial wood products.**

**Figure S65 | Neighbour-joining tree constructed from single DNA barcode region, *ndhF-rpl32*, for species identification of *Pterocarpus* commercial wood products.**

**Figure S66 | Neighbour-joining tree constructed from multi-loci combination ITS2+*matK* for species identification of *Pterocarpus* commercial wood products.**

**Figure S67 | Neighbour-joining tree constructed from multi-loci combination ITS2+*rbcL* for species identification of *Pterocarpus* commercial wood products.**

**Figure S68 | Neighbour-joining tree constructed from multi-loci combination ITS2+*ndhF-rpl32* for species identification of *Pterocarpus* commercial wood products.**

**Figure S69 | Neighbour-joining tree constructed from multi-loci combination *matK*+*rbcL* for species identification of *Pterocarpus* commercial wood products.**

**Figure S70 | Neighbour-joining tree constructed from multi-loci combination *matK*+*ndhF-rpl32* for species identification of *Pterocarpus* commercial wood products.**

**Figure S71 | Neighbour-joining tree constructed from multi-loci combination *rbcL*+*ndhF-rpl32* for species identification of Pterocarpus commercial wood products.**

**Figure S72 | Neighbour-joining tree constructed from multi-loci combination ITS2+*matK*+*rbcL* for species identification of *Pterocarpus* commercial wood products.**

**Figure S73 | Neighbour-joining tree constructed from multi-loci combination ITS2+*matK*+*ndhF-rpl32* for species identification of *Pterocarpus* commercial wood products.**

**Figure S74 | Neighbour-joining tree constructed from multi-loci combination ITS2+*rbcL*+*ndhF-rpl32* for species identification of *Pterocarpus* commercial wood products.**

**Figure S75 | Neighbour-joining tree constructed from multi-loci combination *matK*+*rbcL*+*ndhF-rpl32* for species identification of *Pterocarpus* commercial wood products.**

**Figure S76 | Neighbour-joining tree constructed from multi-loci combination ITS2+*matK*+*rbcL*+*ndhF-rpl32* for species identification of *Pterocarpus* commercial wood products.**

**Figure S77 | Full agarose gel electrophoresis images in Figure 1.**

For labels and figure legends, please refer to Figure 1.

**(1a)**

**(1b)**

**(1c)**

**Figure S78 | Full agarose gel electrophoresis images in Figure 2.**

For labels and figure legends, please refer to Figure 2.

**(2a)**

**(2b)**

**(2c)**

**Figure S79 | Full agarose gel electrophoresis images in Figure 3.**

For labels and figure legends, please refer to Figure 3.

**(3a)**

**(3b)**

**(3c)**

**(3d)**

**(3e)**

**(3f)**

**Figure S80 | Full agarose gel electrophoresis images in Figure 4.**

For labels and figure legends, please refer to Figure 4.

**(4a)**

**(4b)**

**(4c)**

**(4d)**

**(4e)**

**(4f)**

**Figure S81 | Full agarose gel electrophoresis images in Figure 5.**

For labels and figure legends, please refer to Figure 5.

**(5a)**

**(5b)**

**(5c)**

**(5d)**

**(5e)**
